# Astrocyte Enrichment of 3D Cortical Constructs Enhances Brain Repair

**DOI:** 10.1002/advs.202507423

**Published:** 2026-02-26

**Authors:** Elisa M. Cruz, Luana C. Soares, Gretchen Greene, Fernando Messore, Mohammed Abuelem, Mingyu Li, Caroline Andersen, Mirriam Domocos, Elisa Vitiello, Rabeah Abdul Razak, Marlene Lawston, Gabriel Moser, Talia Vasaturo‐Kolodner, Mona Barkat, Roslyn M. Bill, Edward Mann, Linna Zhou, Mootaz M. Salman, Hagan Bayley, Zoltán Molnár, Francis G. Szele

**Affiliations:** ^1^ Department of Physiology, Anatomy and Genetics University of Oxford Oxford UK; ^2^ Aston Institute for Membrane Excellence and School of Biosciences Aston University Birmingham UK; ^3^ Department of Biochemistry University of Oxford Oxford UK; ^4^ Department of Chemistry University of Oxford Oxford UK; ^5^ National Institute of Mental Health NIH Bethesda MD USA; ^6^ National Institute of Neurological Disorders NIH Bethesda MD USA; ^7^ Faculty of Medicine Alexandria University Alexandria Egypt; ^8^ BHF Oxford Centre of Research Excellence University of Oxford Oxford UK; ^9^ Kavli Institute for NanoScience Discovery University of Oxford Oxford UK; ^10^ British Heart Foundation (BHF) ‐ UK Dementia Research Institute (UK DRI) Centre for Vascular Dementia Research at the University of Oxford Oxford UK

**Keywords:** Astrocytes, Brain implantation, Traumatic brain injury, Cerebral cortex development, Microfluidics tissue printing

## Abstract

Regenerative medicine offers a promising approach to treat brain injuries, yet challenges persist in promoting neuronal survival and integration. Recent studies demonstrate that human cells implanted into rodent brains can exhibit plasticity, integrate into neural circuits and alleviate functional deficits. However, integration is often poor, with inadequate vascularization, and insufficient support cells such as astrocytes. Astrocytes play a crucial role in neuronal development and recovery by releasing growth factors, facilitating synaptogenesis, and promoting blood vessel formation. This study investigated human neuronal progenitor cells cultured alone or cultured with mouse astrocytes and formed into 3D constructs using microfluidics. Co‐cultures exhibited enhanced neuronal maturation, viability, and density. Following implantation into mouse brains, co‐cultures reduced lesion size, increased axonal growth, and improved astrocyte coupling to blood vessels within the graft. Additionally, we show that NPCs and co‐cultures increased astrocyte size in implants. Deconvolved high‐resolution microscopy identified synapses and optogenetics showed functional connections between the host and implants. These findings underscore the essential role of astrocytes in enhancing neuronal tissue integration and advancing brain injury treatments.

## Graphical abstract

This study highlights the role of astrocytes in supporting neural progenitor cell survival and differentiation after traumatic brain injury. Astrocytes enhanced neuronal differentiation, improved cell survival in co‐cultures, and promoted integration of microfluidics‐based implants with host tissue following implantation. Additionally, increased axonal projections and vascular functionality observed in the co‐culture group suggest the crucial role of astrocytes in brain repair strategies.

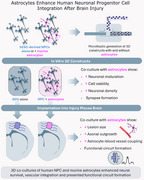

## 1 Introduction

Regenerative medicine has gained significant traction as a therapeutic strategy for brain injuries. However, ensuring neuronal survival and maturation remains a major challenge. A key limitation is the lack of glial support cells, particularly astrocytes, which play essential roles in neuronal development, function, and repair [1, 2]. Astrocytes contribute to neuronal development by secreting neurotrophic factors that promote survival, differentiation, and synaptic formation [1, 2]. In pathological conditions such as Alexander's disease, in which mutations in the gene for glial fibrillary acidic protein (GFAP) lead to astrocyte degeneration, neuronal support is severely compromised [3].

Several pioneering studies have reported that human neuronal progenitor cells (NPCs) implanted into the mouse cerebral cortex can integrate with host tissue and extend axons into surrounding regions [4, 5, 6, 7, 8, 9, 10]. However, most of these approaches lacked support cells and appropriate extracellular matrix, both of which are essential for facilitating robust neuronal connectivity and integration. One recent study added astrocytes to organoids by accelerating the derivation of these crucial cells [11]. Spatial transcriptomics revealed layer‐dependent expression profiles among multiple astrocyte subclasses in organoid transplants [11]. Using an in vivo acute neuroinflammation model, they found a subset of astrocytes that rapidly triggered pro‐inflammatory pathways in response to cytokines [11]. These findings demonstrate the value of 3D culture systems in studying human astrocyte development and function.

The shift from two‐dimensional (2D) to three‐dimensional (3D) cell culture models marks a key advancement in the study of brain development, function, and disease. Traditional 2D cultures fail to replicate the complex spatial and mechanical properties of brain tissue, limiting our understanding of intricate cellular interactions [12, 13, 14]. By contrast, 3D constructs, such as cerebral organoids and bioprinted structures, offer more physiologically relevant models that better mimic the architecture and microenvironment of the brain [15, 16]. These advanced systems hold particular promise for implantation therapies, as they enhance cell‐cell and cell‐matrix interactions, potentially improving engraftment and functional integration with host tissue [16, 17, 18, 19]. Additionally, microfluidics techniques enhance the high‐throughput generation of 3D constructs, improving scalable and efficient approaches for studying neuronal repair and regeneration [20]. Despite these advancements, existing models still struggle to fully replicate the cellular complexity of the developing human brain, and neuronal survival remains a key limitation, often attributable to an insufficient biomimetic microenvironment.

Microfluidics techniques enhance the high‐throughput generation of 3D constructs, improving scalable and efficient approaches for studying neuronal repair and regeneration [20]. This technique can also provide the backbone of other cell culture environments, for example microgrooves that confer mechanical orientation of cell processes and thus aid microcircuitry development [21]. Neurons additionally influence the frequency of astrocytic calcium waves, visualized in vitro [21]. 3D systems can probe astrocytic neuronal interactions in diseases such as tauopathies and allow distinction between cell‐autonomous and non‐cell‐autonomous mechanisms [22]. In an elegant study, Hyvärinen and colleagues demonstrated that co‐stimulation of human cells with inflammatory cytokines induced astrocyte reactivity in vitro [23] showing that astrocyte‐secreted factors can influence neuronal activity [23]. Another study incorporated 3 cell types, astrocytes, neurons and microglia, to model in vitro neuropathological hallmarks of Alzheimer's disease [24]. These studies verified the relevance of microfluidic‐generated 3D models to understand cellular interactions in vitro. However, how these models can be leveraged for applications in regenerative medicine remains unclear.

In our study, we addressed this question by implanting both human NPCs and human NPC/murine astrocyte co‐cultures into the motor and somatosensory cortex of immunosuppressed mice, utilizing either conventional cell suspensions or microfluidics‐generated 3D constructs. Notably, the inclusion of astrocytes enhanced neuronal proliferation and differentiation post‐implantation, demonstrating their essential role in promoting recovery after traumatic brain injury (TBI). The 3D co‐culture constructs not only improved integration with host tissue but also significantly contributed to the healing process. Moreover, astrocytes within the implants increased physical interactions with blood vessels, further supporting neuronal survival and maturation. These findings reinforce the crucial role of astrocytes in regenerative therapies and provide valuable insights for optimizing future strategies aimed at neuronal tissue engraftment and functional recovery. Our 3D microfluidics‐generated model shares several features with other 3D cultures such as 3D bio‐printing [16], yet it is simpler and therefore more accessible to scientists.

## 2 Results

### 2.1 Murine astrocytes in Co‐Cultures Increase Maturation of Human Neurons in Vitro and in Vivo

**FIGURE 1:**
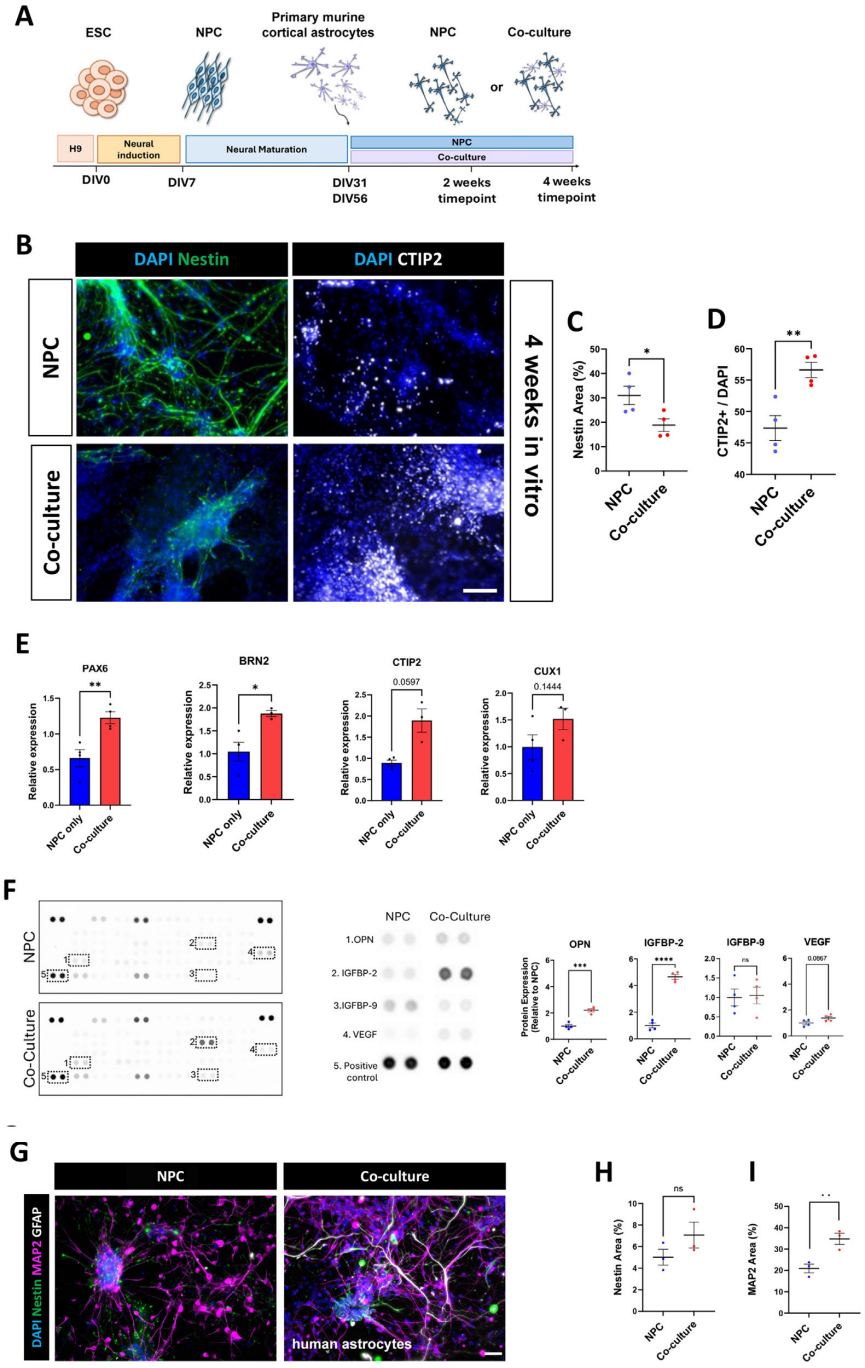
Co‐culture with astrocytes increases the maturation of neurons in monolayer cultures. (A) Schematic of 2D monolayer culture. NPC and co‐cultures were collected for immunofluorescence analysis 2 or 4 weeks after astrocytes were added. (B) Immunofluorescence of progenitor (Nestin) and neuronal marker (CTIP2)in both groups at 4 weeks. Scale bar=100 µm. (C) Nestin immunofluorescence was significantly decreased in the Co‐culture group compared to the NPC group at 4 weeks. The graph displays the mean from two biological repeats across four technical replicates, with statistical analysis performed using an unpaired t‐test (p < 0.05). (D) The percentage of DAPI+ nuclei that were CTIP2+ was increased at 4 weeks in the o‐culture group. Two biological repeats across four technical replicates. Unpaired t‐test. p < 0.01. (E) qPCR analysis for neurodevelopmental transcription factors in 2D cultures. Cells were cultured for 2 weeks, and the data was normalized to the number of neurons in the co‐culture. Both Pax6 and BRN2 were significantly increased in the co‐culture group. CTIP2 and CUX1 expression trended towards greater values than in the NPC group but the results were not significantly different. The graph presents the mean from n=3 biological replicates, analyzed using an unpaired t‐test (*p<0.05, ** p<0.01). (F) Dot blots of angiogenesis array of conditioned media from NPCs and co‐cultures. Osteopontin and IGFBP‐2 are increased in the co‐culture conditioned media. The graph presents the mean from n=3 biological replicates, analyzed using an unpaired t‐test (p < 0.05, ns=non‐significant, ***p<0.01, ***p<0.001). (G) Nestin, MAP2 and GFAP immunofluorescence NPC compared with NPC/human astrocytes in 2D co‐culture. Cells were cultured for 2 weeks, note the GFAP+ astrocytes. Scale bar=50 µm (H) Quantification of percentage of area covered by Nestin did not reveal significant changes. The graph displays the mean from (n = 4) biological experiments, with statistical analysis performed using an unpaired t‐test (ns=non‐significant). (I) MAP2 immunofluorescence surface area was significantly increased in the co‐culture compared to the NPC group. The graph displays the mean from (n = 4) biological experiments, with statistical analysis performed using an unpaired t‐test (p < 0.05).

We postulated that primary murine astrocytes cultured with human embryonic stem cell (hESC) derived neural progenitors (NPCs) (co‐cultures) would increase NPC viability and neuronal differentiation. Therefore, hESC‐derived human cortical NPCs were either grown alone or cultured together with murine astrocytes for 2 or 4 weeks in two‐dimensional (2D) monolayer cultures (Figure 1A). We used a ratio of 1:3 astrocytes to NPCs for the co‐culture group and grew them in neural maintenance medium (NMM). Astrocytes survived well in the NMM co‐cultures and maintained GFAP and S100b expression throughout the duration of the culture (Figure S1A). At 4 weeks in vitro, expression of the neural stem and progenitor protein Nestin decreased when cells were in co‐cultures (Figures 1B,C), indicating that astrocytes may have promoted cell lineage progression. At the same timepoint, the number of nuclei positive for the deep neuron layer marker CTIP2 was greater in co‐cultures compared to the NPC's alone (Figures 1B,D). This suggested that murine astrocytes promote human neuronal differentiation.

We next asked if neurodevelopmental transcription factors were altered in the co‐cultures compared to NPCs (Figure 1E). Using qPCR, we analyzed expression of PAX6, BRN2, CTIP2 and CUX1. PAX6 is mainly expressed by stem and progenitor cells and is necessary for cortical development [25]. Later‐born, upper‐layer neurons can be detected by BRN2 or CUX1 expression, whereas CTIP2 expression identifies early born, deep layer neurons [26]. Our results showed an approximate 2‐fold increase in the expression of PAX6 and BRN2 in the co‐culture group, while there was no significant difference in CTIP2 and CUX1 expression between the two groups (Figure 1E). Together with the immunofluorescence quantification, this data suggests that at 2 weeks neuronal differentiation is increased in the co‐culture group compared with the NPC group.

Co‐cultures could alter the expression not only of intrinsic factors but also of secreted molecules, thereby increasing neuronal maturation. To test this, we treated NPCs with conditioned medium that had been collected from co‐cultures for 2 weeks. Comparison of the percentage of Nestin+ and MAP2+ areas in the NPC only (NMM medium) and NPC‐treated with conditioned medium showed no significant difference at 2 weeks (Figure S1B). This matches results showing Nestin and MAP2 expression in NPC only and co‐culture groups at 2 weeks at the same time point (Figure S1C).

Survival and integration of implants depend largely on angiogenesis and perfusion of the 3D structures post‐implantation. Therefore, we collected conditioned media from both groups and performed an antibody‐based angiogenesis dot‐blot array as described previously [27] (Figure 1F). The co‐culture medium showed a significant increase in the expression of Osteopontin (OPN) and Insulin‐like Growth Factor Binding Protein 2 (IGFBP‐2) (Figure 1F), which are related to brain development and plasticity [28, 29, 30]. We saw no upregulation of Vascular Endothelial Growth Factor (VEGF), which is a regulator of vasculogenesis and angiogenesis [31], nor of Insulin‐like Growth Factor Binding Protein‐9 (IGFBP‐9), a matricellular protein which is involved in brain development and disease [32].

While examining the morphology of the neuronal maturation marker MAP2, we noticed serial swellings in some MAP2+ processes (Figure S1D), which can be indicative of axonal degeneration [33, 34, 35]. Axonal degeneration leads to cell death during development and in disease and can be observed in vitro in a variety of systems, its hallmark being serial swelling of neurites [33, 34]. Other work showed neurite damage and cell death in cortical neurons derived from hiPSCs [35]. Therefore, we quantified the percentage of degenerating MAP2+ neurites as a readout of neuron health (Figure S1E). The astrocyte co‐culture condition reduced the number of degenerating neurites at 2 and 4 weeks of culture (Figure S1E), suggesting that astrocytes decrease degeneration.

Many in vivo studies, notably from the Gaillard and Vanderhagen groups, have demonstrated that implanted single cell suspensions survive in the host cerebral cortex, mature and integrate into host circuitry [4, 5, 6, 7, 8, 9, 10]. Using protocols similar to the above studies, we grew NPCs alone or in the presence of astrocytes from day 19 until day 31of the neural maturation protocol [36]. co‐cultures were carried out prior to cell injections in order to assess their beneficial effects on NPC survival and maturation after brain injection. We injected 100,000 cells in liquid suspension into the motor cortex of immune‐suppressed NOD‐SCID gamma (NSG) mice at postnatal day 8 (P8) (Figure S2A). We qualitatively examined neuronal maturation of single cell injections and found robust HuNu, NeuN and MAP2 expression (Figure S2B) suggesting that the injected cells survived and differentiated. We next examined the percentage of HuNu cells that were maturing, proliferating, or exhibiting cell death (Figure S2C–F). Dense clusters of small but clearly delineated HuNu+ cells were found and some of these expressed low levels of the pan‐neuronal marker NeuN, suggesting they were starting to mature (Figure S2C). The adjacent mouse cortex contained large NeuN+ neurons, which served as an internal positive control. Interestingly, the percentage of HuNu+ cells that were NeuN+ was greater in the co‐culture group (Figure S2D), suggesting augmented maturation. As expected, based on the heterogeneity of the cell suspension, we also found some Ki67+ proliferating cells in the implants. Since we had shown that astrocytes were promoting lineage progression and neuronal maturation in vitro, we expected to see fewer mitotic cells in the co‐culture implants. However, we found the opposite, co‐cultures had a significantly higher percentage of HuNu+ cells that were Ki67+ (Figure S2E). We next examined activated caspase‐3 as a marker of apoptosis and showed minimal programmed cell death in both groups (Figure S2F). The combination of our in vitro and in vivo experiments supported our core hypothesis that astrocytes would induce neuronal differentiation in co‐cultures.

Human astrocytes can differ from murine astrocytes, for example in size [37, 38, 39, 40, 41, 42, 43], and it was unclear if their actions on co‐cultures would be similar. We co‐cultured NPCs with human astrocytes (ScienCell) for two weeks and analyzed them in vitro (Figure 1G). We seeded the same overall number of cells as in the NPC/murine astrocyte experiments and at the same ratios. Immunofluorescence showed that the surface area occupied by Nestin+ immunofluorescence was not significantly different compared to NPC (Figure 1H). However, the expression of MAP2 (Figure 1I) was significantly greater in the NPC/human astrocyte group than in the NPC group alone. This suggested human astrocytes can also increase neuronal maturation.

### 2.2 Co‐Cultures Enhance Viability and Maturation in Microfluidics‐Generated 3D Constructs in Vitro

**FIGURE 2:**
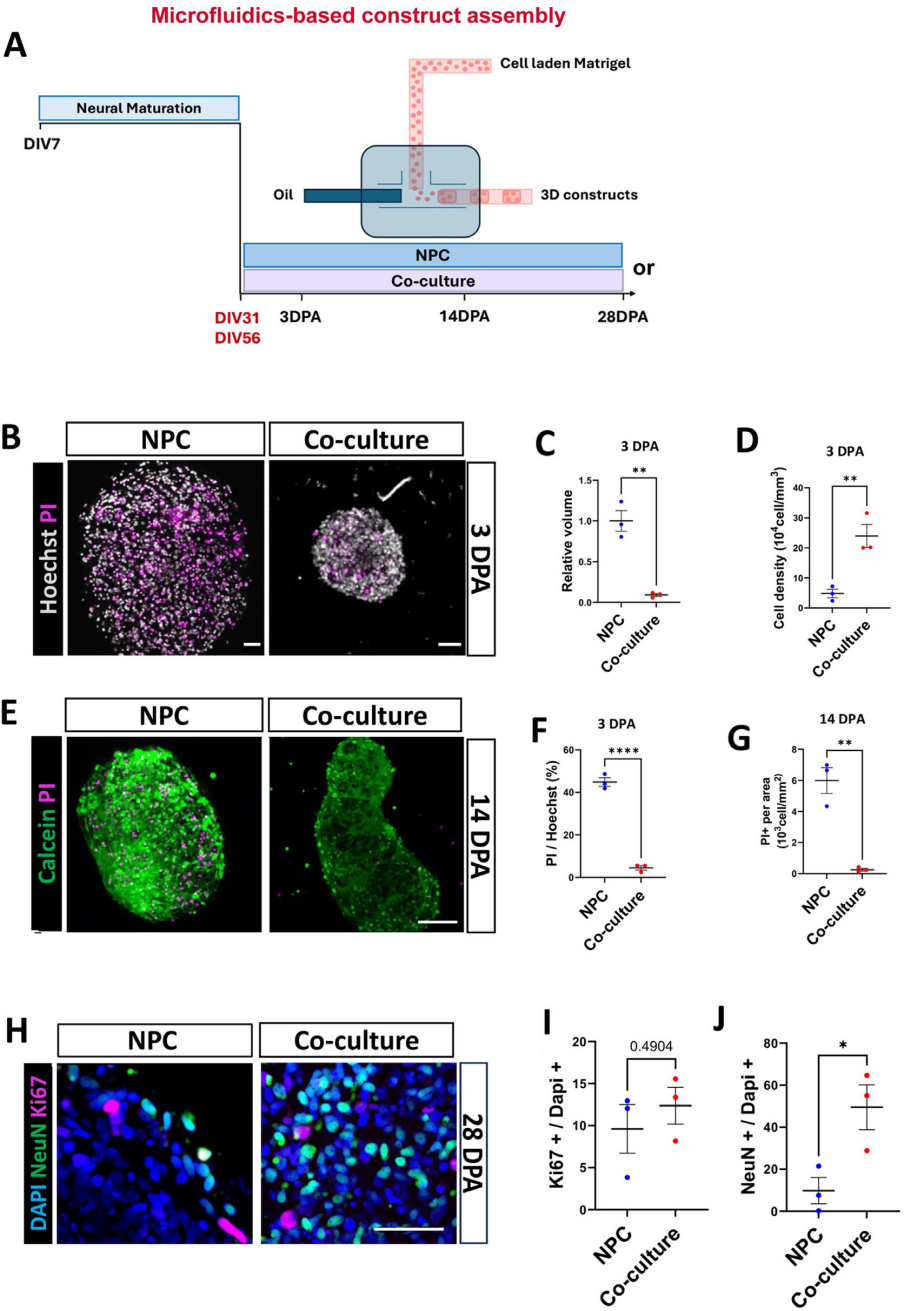
Co‐cultures enhance viability and maturation in microfluidics‐generated 3D constructs. (A) Schematic representation of our microfluidics device featuring two inlets and one outlet for cell culture assembly. Both murine astrocytes and neuronal progenitor cells (NPCs) were harvested simultaneously and mixed during construct assembly. (B) At 3 days post‐assembly (DPA), co‐cultured constructs (NPC/astrocyte) were smaller than NPC constructs. Nuclei are stained with Hoechst (grey), with dead cells highlighted by propidium iodide (PI) (magenta). The NPC/astrocyte constructs appear more cell dense. Scale bar=50 µm. (C) Construct volume at 3 DPA was significantly smaller in the co‐culture group compared to the NPC group. Data represent the mean of three biological replicates (n=3). Statistical significance was assessed using an unpaired t‐test (p < 0.01). (D) Cell density was significantly higher in the NPC/astrocyte co‐culture compared to the NPC group. Data represent the mean from three biological replicates (n=3). Statistical analysis was performed using an unpaired t‐test (p < 0.01). (E) A live/dead assay in the NPC only and in the NPC/astrocyte co‐cultures at 14 DPA. Live cells are visualized using Calcein (green), whereas dead cells are indicated by PI. Scale bar=50 µm. (F) Decreased cell death, indicated by percentage of PI+/ Hoechst+, observed in the co‐culture group relative to the NPC group at 3 DPA. Data represent the mean of three biological replicates (n=3). Statistical significance was determined using an unpaired t‐test (p < 0.0001). (G) Decreased cell death, indicated by the density of PI, was observed in the co‐culture group relative to the NPC group at 14 DPA. Data represent the mean of three biological replicates (n=3). Statistical significance was determined using an unpaired t‐test (p < 0.0001). (H) Immunofluorescence of NeuN and Ki67 in 3D constructs at 28 DPA. Scale bar=50 µm. (I) The proportion of proliferating cells (Ki67+/DAPI+) at 28 DPA was similar in both NPC and Co‐culture groups. Data are from three biological replicates (n=3). P‐values are provided following unpaired t‐test analysis. (J) The proportion of mature neurons (NeuN+/DAPI+) at 28 DPA was greater in the co‐culture group compared to the NPC group. Data are from three biological replicates (n=3). Statistical significance was assessed using an unpaired t‐test (p < 0.05).

We next used a microfluidic system to create three‐dimensional constructs. As with the experiments above, we differentiated NPCs into cortical neurons following an adapted version of the Shi et al., (2012) protocol [36, 44]. 3D constructs were produced with a bespoke microfluidics device containing a three‐way tube system with two inlets and one outlet connected by a T‐junction (Figure 2A) One inlet was loaded with bio‐ink, cells resuspended in Matrigel, another inlet was loaded with undecane oil, and the outlet collected cell‐laden Matrigel droplets as 3D constructs (Figure 2A). The flow rate of oil and bio‐ink and the diameter of the collection tubing defined the dimensions of the constructs. We first tested the method by assembling 3D constructs containing murine astrocytes only. Cells in the construct at 5 days and 2 and 4 weeks post‐assembly were GFAP+ and S100β+ even though they were grown in neural maintenance medium (Figure S3A). Quantification of the positive area of Nestin within 3D microfluidic‐generated constructs showed no significant difference between NPC only and co‐culture groups at 14 DPA (days post‐assembly) (Figure S3B,C). We then generated NPC and co‐culture constructs. NPCs or NPC/astrocytes (3:1) were resuspended in Matrigel at a final density of 8x10^7^ cells/mL [16].

The two groups of NPC constructs were similar in size upon generation, but, unexpectedly at the early stage of 3 DPA, many of the NPC constructs were significantly larger than the co‐culture constructs (Figure 2B, C; Figure S4A,B). Even though the co‐culture constructs were smaller, they had a higher cell density, as measured by the number of Hoechst‐stained cells per unit volume using Z‐stacks and confocal microscopy (Figure 2D). At the later time points of 14 and 28 DPA, there were no statistically significant difference in volume between the co‐culture and NPC groups (Figure S4C, D). This could be partially explained by the increased growth index (relative to 3 DPA) in co‐culture constructs at 14 DPA (Figure S4C, E). We predicted that the greater concentration of cells in the co‐culture constructs may be due to increased proliferation, in the constructs with astrocytes, but this was not supported, since the percentage of Ki67+/DAPI+ nuclei was not different between the groups at 14 and 28 DPA (Figure 2H,I; Figure S4F,H).

Microfluidics‐generated 3D constructs of NPCs alone and co‐cultures both survived well at 3 and 14 days post‐assembly (DPA) as indicated by propidium iodide (PI) staining. Interestingly the co‐culture constructs had significantly fewer PI+ dead cells (Figure 2B,E,F,G). Some of these cells had neurites emanating from them, suggesting neuronal differentiation (Figure 2E). Mature neurons were observed in the constructs via NeuN immunostaining at 14 and 28 DPA (Figure 2H, J; Figure S4F,G). At 28 DPA, the co‐culture constructs had a higher number of NeuN+ cells (Figure 2J). This supports the notion that astrocytes induce neuronal maturation in 3D cultures, as seen in 2D cultures. Taken together, these data show that, compared to NPC alone, murine astrocytes in co‐cultures increased human cell survival and neuronal differentiation of microfluidics‐generated 3D constructs in vitro.

### 2.3 Astrocytes Promote TBI Lesion Recovery Upon Implantation of Co‐Cultures

**FIGURE 3:**
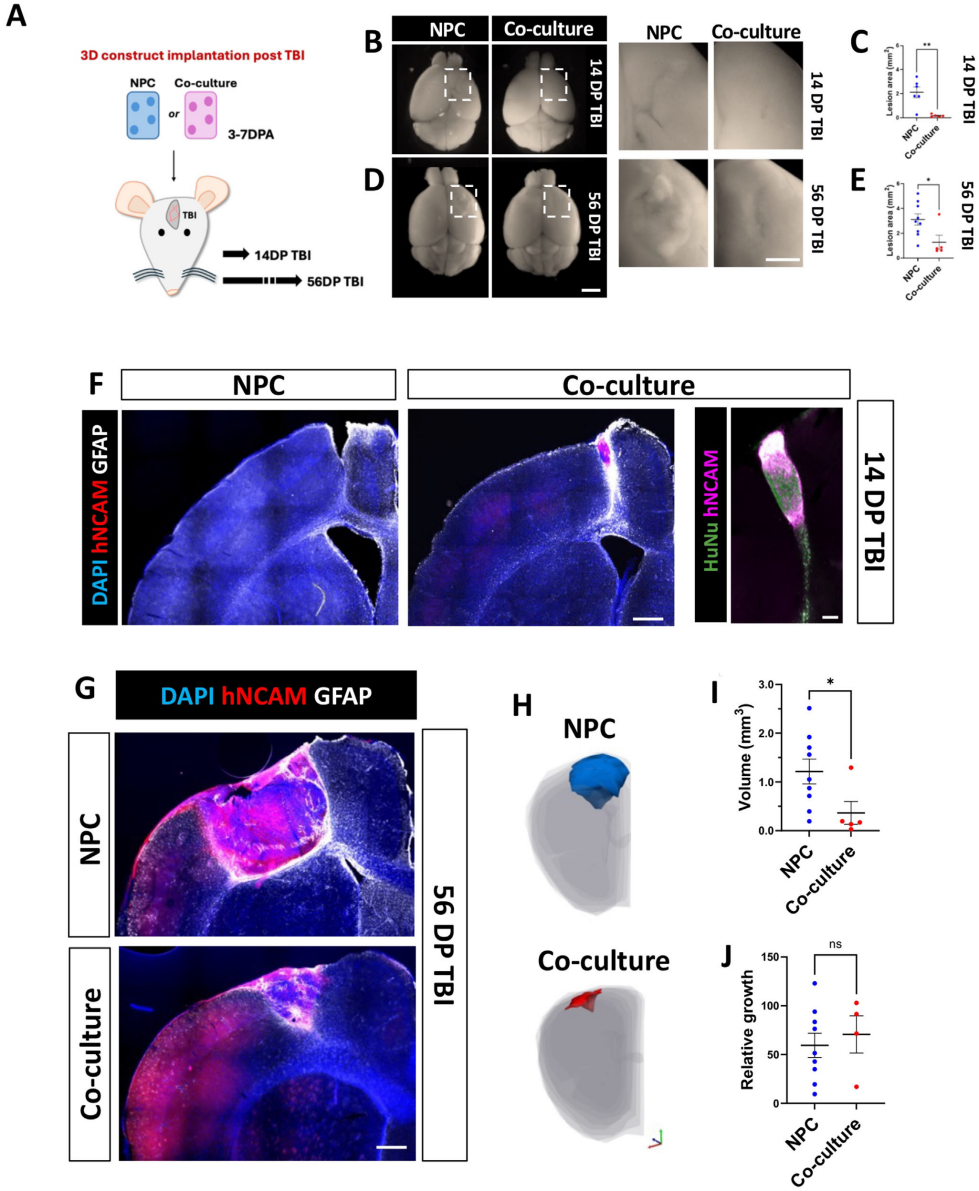
Astrocytes promote TBI lesion recovery upon implantation of co‐culture constructs. (A) Schematic depicting the implantation of 3D constructs into the brain following traumatic brain injury (TBI). 3–7 days‐post‐assembly (DPA,) TBI was carried out and constructs immediately implanted. Brains were collected at 14 or 56 days post TBI (DP TBI). (B) Representative images of whole brains collected at 14 DP TBI. White squares delineate the region of the lesion (scale bar=2 cm). Lesions are highlighted in the left panels (scale bar=1 cm). (C) Quantification of visible lesion area illustrated in panel B. The graph displays the mean lesion area across six biological replicates for the NPC group and four for the co‐culture group. Statistical differences were evaluated using an unpaired t‐test (p < 0.01). (D) Representative images of whole brains collected at 56 DP TBI (scale bar=2 cm). The lesion areas are highlighted in white squares and shown in the left panels (scale bar=1 cm). Lesions remained less prominent in the co‐culture group. (E) Quantification of the visible lesion area measured at 56 DP TBI. The graph shows the mean across nine mice for the NPC group and five for the co‐culture group. Statistical analysis was conducted using an unpaired t‐test (p < 0.05). (F) Representative immunofluorescence of coronal brain sections at 14 DP TBI from mice receiving NPCs or co‐culture implants. Scale bar=500 µm in lower magnification and 100 µm in high magnification panel showing details of the co‐culture implants. (G) Representative immunofluorescence of hNCAM in implants at 56 DP TBI from mice that received implanted NPC or co‐culture constructs. Scale bar=500 µm. (H) Example of implant tracing for volume measurement on Neurolucida software for both groups. (I) Volumes of implanted of NPC and co‐culture implants at 56 DP TBI. Note that co‐culture implants were smaller. Statistical analysis was conducted using an unpaired t‐test (p < 0.05). (J) There were no significant differences in the relative growth (size change from 3 DPA to 56 DP TBI) in the two groups to their volumes at 3DPA. Statistical analysis was conducted using an unpaired t‐test (p < 0.05) (ns=non‐significant).

The above findings indicated that astrocyte co‐cultures may enhance implantation success. To investigate this, we analyzed lesion healing and neuronal survival following implantation of microfluidics‐generated 3D constructs into the cerebral cortex of postnatal NSG mice. We first generated NPC and co‐culture constructs as described above and allowed them to recover for 3‐7 DPA. A unilateral aspiration traumatic brain injury (TBI) was induced in the motor and somatosensory cortex [45, 46] of postnatal (P7‐P9) mice, and a construct was immediately implanted into the lesion (Figure 3A). The brains of the implanted mice were collected at either 14 or 56 days post‐TBI (DP TBI).

Gross morphological analysis of the brains showed distinct lesions in the NPC implantation group at 14 days post‐TBI (14 DP TBI), while the co‐culture group had minimal lesions (Figure 3B). Quantification of lesion size revealed a statistically significantly smaller lesion size in the co‐culture group compared to the NPC group (Figure 3C). At 56 DP TBI, the lesion appeared to be resolved in both groups; though the NPC group still displayed visible scarring in the TBI area, while the co‐culture group a smoother cortical surface in the TBI area (Figure 3D). Similar to the 14 DP TBI timepoint, the lesion area was smaller in the co‐cultures compared to the NPC at 56 DP TBI (Figure 3E).

We next examined the implanted microfluidic‐generated constructs at the cellular level with human‐specific antbodies against human neural cell adhesion molecule (hNCAM) and human nucleus (HuNU). We found no implanted hNCAM+ cells in the NPC group after 14 days (Figure 3F). In contrast, the co‐culture implants displayed clear hNCAM+ and HuNu+ cell clusters at the same time point. By 56 DP TBI, the NPC group had large lesions filled with hNCAM+ cells and a distinct GFAP+ astrocytic border (Figure 3G). As noted in our gross anatomical observations, the TBI lesions and implanted constructs were significantly smaller in the co‐culture group compared to the NPC group at 56 days (Figures 3H, I). However, when normalized to the average construct size at 3 DPA in vitro, the groups showed no difference in volume increase, indicating that they expand at the same rate (Figure 3J). These findings highlight the significant role of astrocytes in enhancing the repair of large TBIs when added to NPCs.

### 2.4 Implantation Increases Astrocyte Size and Complexity

**FIGURE 4:**
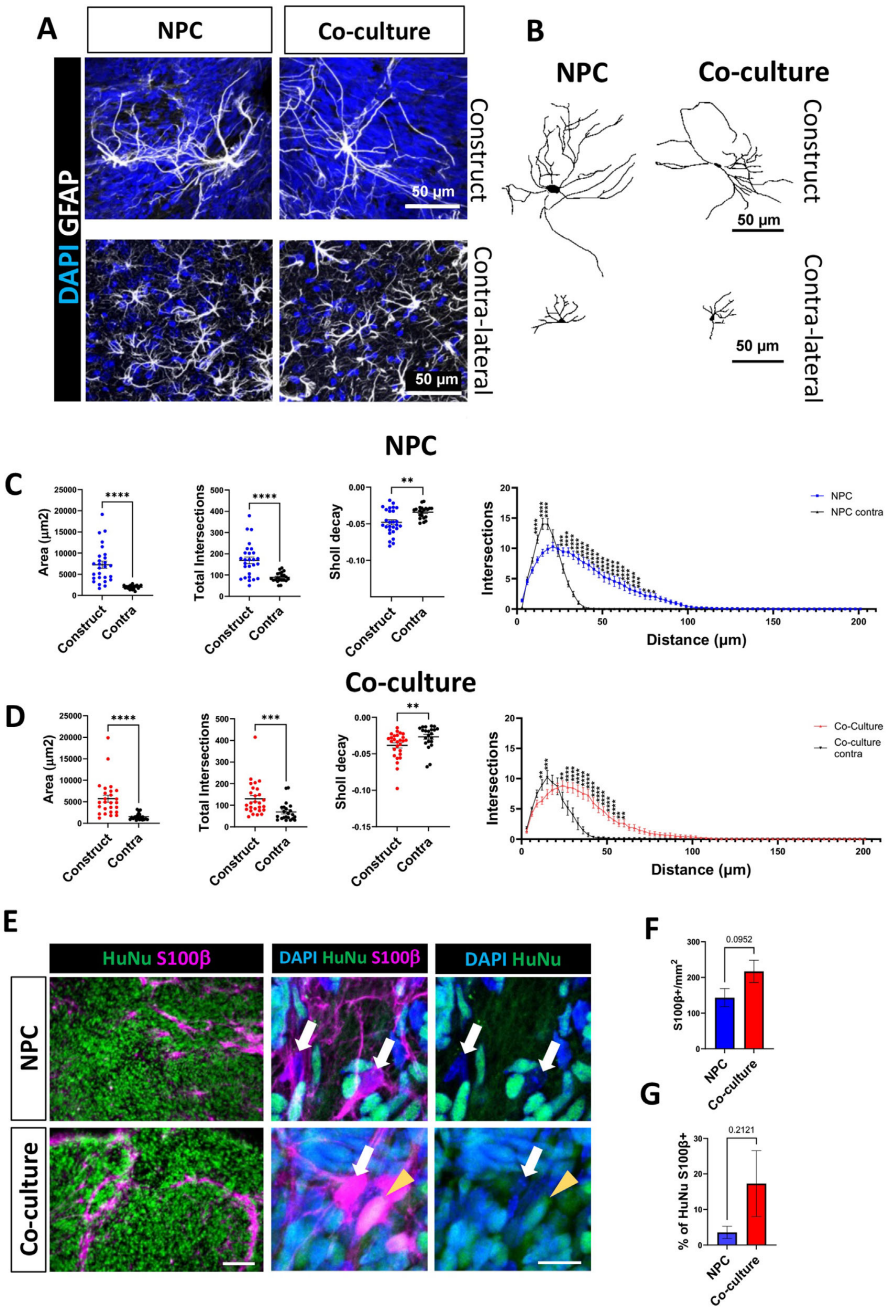
Implantation increases astrocyte size and complexity. (A) Representative confocal Z‐projections of astrocytes identified with GFAP immunofluorescence in the constructs as well as in the contralateral hemispheres at 56 DP TBI. Scale bar=50 µm. (B) Reconstructions of astrocyte skeletons using Neurolucida software based on GFAP images from panel A. Scale bar=50 µm. (C, D) Sholl analysis for astrocytes in the NPC (C) and co‐culture (D) groups at 56 DP TBI. Astrocytes located within the constructs were larger and had a higher total number of intersections compared to contralateral cortex astrocytes within the same group. Additionally, the Sholl analysis revealed an increased number of intersections with increasing distance from the cell body in astrocytes from the constructs. We observed a smaller slope index for construct‐derived astrocytes relative to their contralateral counterparts, indicating greater structural complexity. Graphs represent means across 27 cells from 5 animals for both groups, with statistical significance assessed using an unpaired t‐test (***p < 0.001; ****p < 0.0001). (E) Representative immunofluorescence at 56 DP TBI of human S100β+ cells in implanted constructs. Higher magnification images are maximum projections. A rare human astrocyte (S100β+ HuNu+) is indicated with a yellow arrowhead. Murine astrocytes (HuNu‐ S100b+) cells are shown with white arrows. Scale bar =100 µm (smaller magnification) and 15 µm (higher magnification). (F) Quantification of number of S100β+ cells per mm^2^ within the graft at 56 DP TBI. Graphs are mean with SEM. Unpaired t‐test. (p=0.0952). (G) Quantification of the percentage of S100β+ HuNu+ double labelled astrocytes in the implant. Graphs are mean with SEM. Unpaired t‐test. (p=2121).

Several studies have demonstrated that human astrocytes are larger than those in other mammalian species [38]. This difference is maintained when human astrocytes are implanted into the mouse brain. However, it is unknown if murine astrocytes are capable of adopting a larger size, and therefore we addressed this in implanted co‐cultures. We examined astrocyte morphology  based on GFAP expression. This revealed that astrocytes in both NPC and co‐culture implanted constructs were larger than those in the contralateral cortex (Figure 4A). The GFAP+ cells in the implants had long, thin processes and were similar to fibrous rather than protoplasmic astrocytes (Figure 4A). In contrast, the shape of astrocytes in the contralateral cerebral cortex was more protoplasmic—they had shorter and wider processes. To quantify astrocyte morphology, we traced them using Neurolucida (Figure 4B). Astrocytes within the constructs of both the NPC and co‐culture groups exhibited significantly llarger surface areas, perimeters, and increased complexity, as indicated by the increased total number of intersections, the number of intersections per distance and the decay of the Sholl curve, compared to the corresponding contralateral side of the mouse brain (Figures 4C, D). However, there were no differences in most of these parameters between astrocytes in NPC constructs and those in co‐culture constructs (Figure S5A(. There was an increase in the Sholl decay in the co‐culture group (Figure S5B). Notably, contralateral astrocytes in the co‐culture group were slightly but significantly smaller and less complex than those in the NPC implants (Figure S5B).

To evaluate if there was a difference in astrocyte density in the implants between the two groups, we measured the surface area positive for S100β, an established marker of mature astrocytes [47, 48]. Recognizing that astrocytic populations in different brain regions can variably express GFAP, S100β, or both [49, 50], we calculated the Manders co‐localization coefficients which indicate the fraction of GFAP overlapping S100β (M1) and of S100β in GFAP (M2) (Figure S5C). Maximum projection images were generated after image acquisition with a 20x objective, with a pixel size of 0.46 x 0.46 µm. We observed that the average M1 in the NPC and co‐culture groups was 0.81±0.06 and 0.85±0.06 and the average of M2 was 0.72±0.13 and 0.85±0.06, respectively. This indicates a high fractional overlap of both immunofluorescent markers, and the robustness of our data. A similar density of S100β+ astrocytes was seen in NPC and co‐culture groups (Figures 4E,F).

We presumed that similar to our previous work, the NPC implants would contain very few human astrocytes upon implantation [16]. However, it was necessary to assess the origin of the implant astrocytes, as astrogenesis and neurogenesis can occur in the same pool of neural stem cells in a time‐dependent manner [51, 52], suggesting the possibility that the astrocytes were generated by the human NPCs in the constructs. To address this, we quantified the number of S100β+ astrocytes that were also positive for HuNu. This analysis revealed that the astrocytes in the implants of the NPC group were primarily of host murine origin, with the number of human astrocytes in the implants being statistically similar between the two groups (3.54±4.50% in NPC and 17.28±20.71% in co‐culture groups) (Figure 4G).

Our findings demonstrate that astrocytes within both NPC and co‐culture implants exhibit increased size and complexity compared to those in the contralateral cortex, although no significant differences were observed between the two implant groups. Additionally, the astrocytes in NPC implants were found to be primarily of murine origin. Also, the majority of astrocytes in the co‐culture were HuNu negative, as expected, as they contained murine astrocytes. Overall, our result indicates that murine astrocytes increased in size and complexity when implanted with human NPCs in a model of TBI. We speculate that they may be modified by adhesion or secreted molecules expressed in the TBI and/or human NPC implant.

### 2.5 Enhanced Astrocyte Coupling to Blood Vessels in co‐Culture Implants

**FIGURE 5:**
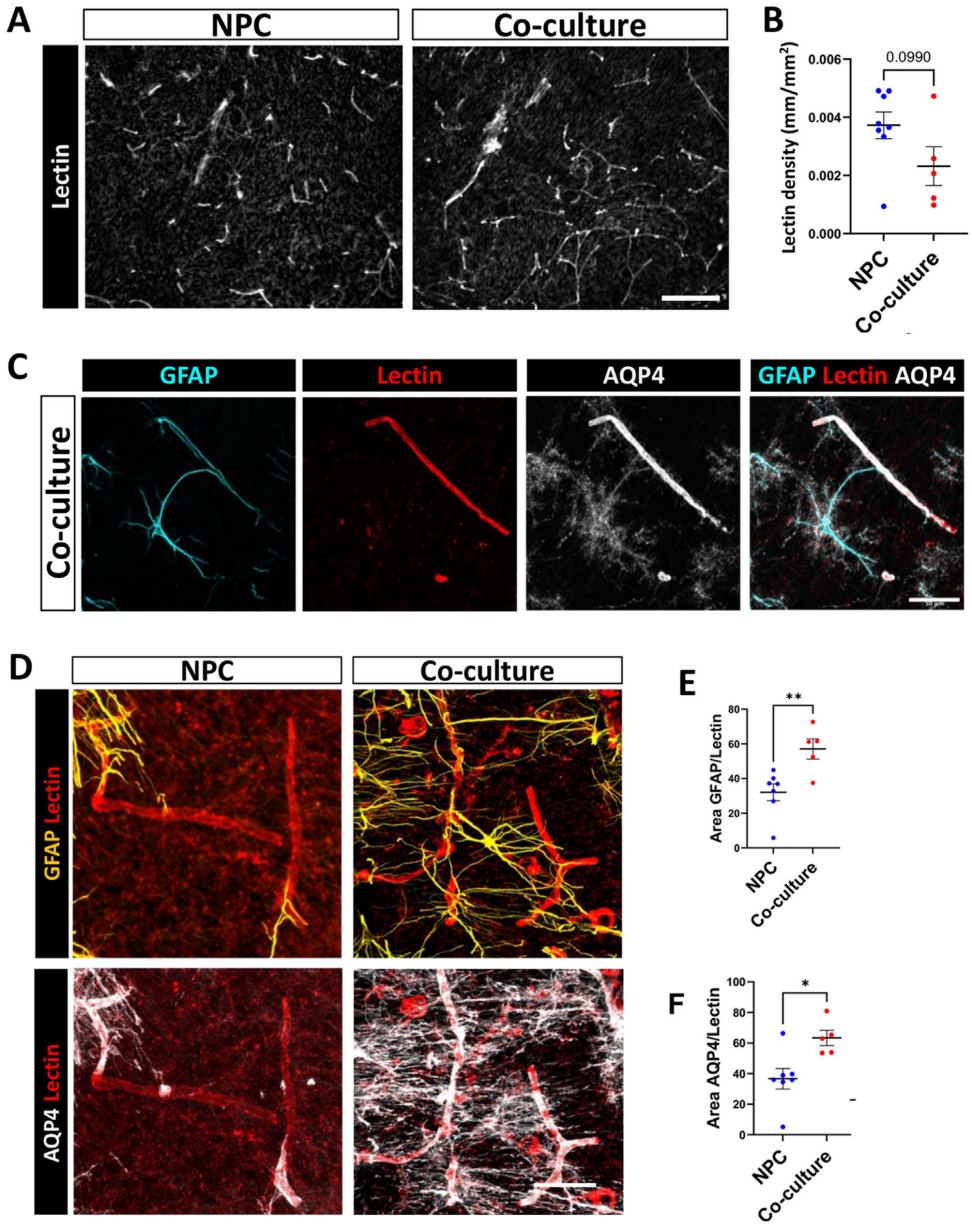
Co‐culture promotes blood vessel association with astrocytes in implanted constructs. (A) Representative image of blood vessels detected with lectin in NPC only and co‐culture constructs 56 DP TBI. Scale bar=200 µm. (B) Quantification of total length of lectin per mm^2^ in NPC only and co‐culture implants 56DP TBI. Graph represents means across 8 animals for the NPC only group and 5 animals for the co‐culture group. Statistical significance assessed using an unpaired t‐test (p=0.0990). (C) Representative labelling of a BV in a co‐culture implant. Note its ensheathment by a GFAP and AQP4+ astrocyte. Scale bar=50 µm. (D) Immunofluorescence in representative images for GFAP and AQP4 associated lectin‐positive BV in NPC only and co‐culture implants at 56 DP TBI. Scale bar=50 µm. (E) Quantification of co‐localization of GFAP and AQP4 with lectin. We observed increased lectin area positive for GFAP and for AQP4 in the co‐culture group compared with the NPC only group. Graph represents means across 8 animals in NPC group and 5 animals in the co‐culture group, with statistical significance assessed using an unpaired t‐test (**p < 0.01). (F) Similarly, compared to NPC, co‐culture implants presented an increased percentage of lectin area also positive for AQP4. Graph represents means across 8 animals in NPC group and 5 animals in the co‐culture group, with statistical significance assessed using an unpaired t‐test (*p < 0.05).

Astrocytes support the development of blood vessels (BV), regulate the blood‐brain barrier (BBB), and promote angiogenesis after injury [53]. Angiogenesis is crucial for the survival and integration of implanted cells and organoids [54], and many preclinical trials have failed due to inadequate perfusion over time. We hypothesized that co‐cultures would enhance angiogenesis in the implants, so we examined BVs using lectin labelling. Analysis of blood vessel abundance within the implants revealed similar levels of angiogenesis in both the NPC and co‐culture groups (Figure 5A, B). However, qualitative analysis revealed a notable difference in the association of BV with astrocytes between the two groups. In the co‐culture group, astrocytes were observed in large numbers near BVs, with their processes ensheathing them (Figure 5C). Additionally, aquaporin‐4 (AQP4), a water channel highly expressed in astrocytic endfeet at the BBB [55], was found in close association with lectin‐positive BVs (Figure 5C).

To assess the structure of the gliovascular interface within the implants, we first examined the coupling between astrocytes and BVs. Quantification of lectin co‐labelled with GFAP, revealed a significantly greater association of BVs with astrocytes in the co‐culture group compared to the NPC group (Figure 5D, E). Similarly, co‐localization of lectin and AQP4, demonstrated that the co‐culture group had significantly more AQP4 coverage of lectin‐stained blood vessels (Figure 5D, F). AQP4 plays fundamental roles in the BBB, being the main water channel in the brain [56]. AQP4 expression is localized throughout the cytoplasm during astrocyte differentiation but it becomes more specifically localized to astrocytic endfeet as the cell matures [56], indicating functionality of the cellular interface of the BBB. Together, these data suggest that the presence of astrocytes in the constructs leads to an increased association of astrocytes with BVs and astrocyte endfoot coverage of the vessels. It also increases AQP4 expression and BV association after implantation, which could result in improved perfusion and BBB function in the implanted tissue.

**FIGURE 6:**
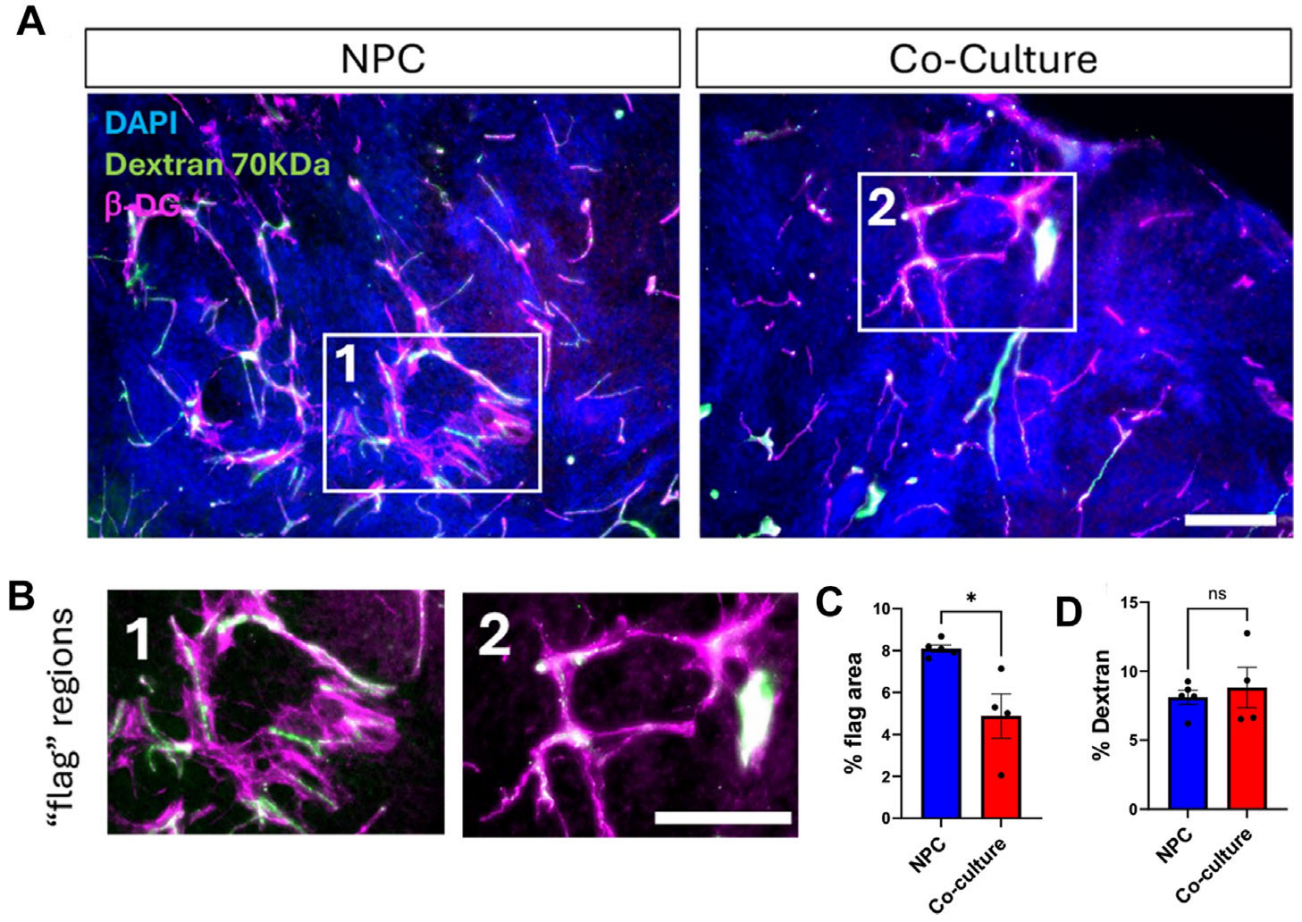
Implanted NPC constructs exhibit abnormal β‐dystroglycan distribution. (A) Blood vessels in implants. β‐dystroglycan immunofluorescence (Magenta) co‐localized with Dextran 70 kDa dextran labelling with FITC (green). Scale bar=200 µm. (B) Insets 1 and 2 from NPC and co‐culture implants, respectively. Note that the flag regions are associated with blood vessels. (C) The area covered by flags was signifcantly smaller in the co‐cultures. 5 animals in NPC group and 4 in co‐culture group, statistical significance assessed using an unpaired t‐test (*p < 0.05). (D) The percentage of area labelled by Dextran was not different between the two groups. 5 animals in NPC group and 4 in co‐culture group, statistical significance assessed using an unpaired t‐test (ns=non‐significant).

β‐dystroglycan is essential for structural integrity and function of the BBB. It is localized at the astrocytic endfeet, where it connects the cytoskeleton to the extracellular matrix and participates in the anchoring of AQP4 [57, 58, 59]. β‐dystroglycan immunohistochemistry in two months post‐TBI NPC implants revealed blood vessels resembling normal capillaries and some larger vessels (Figure 6A). Unexpectedly, we also uncovered networks of β‐dystroglycan+ cellular elements associated with BVs but resembling sheet‐like structures that we term “flags” (Figure 6A). All sections expressed β‐dystroglycan+ flags but there were far fewer in the co‐cultures compared to the NPC implants. Quantitative analysis showed significantly smaller β‐dystroglycan “flag” areas per construct in co‐culture when compared to NPC implants (Figure 6B‐C).

Taken together, these data suggested that there may be differences in BBB leakiness between the two groups. To address this question, we injected 70 KDa dextran labelled with fluorescein via the tail vein into mice at 2 months post‐TBI. We found fluorescein‐tagged dextran inside the blood vessels of both groups at similar densities (Figure 6A,D). This data shows that BVs in implants have integrated with the host vasculature and are irrigated. Minimal fluorescence emanating from blood vessels was observed in either of the groups, suggesting the vasculature in the implant was largely intact and not leaky.

### 2.6 Increased Graft‐Host Anatomical Integration Upon Co‐Culture Implantation

**FIGURE 7:**
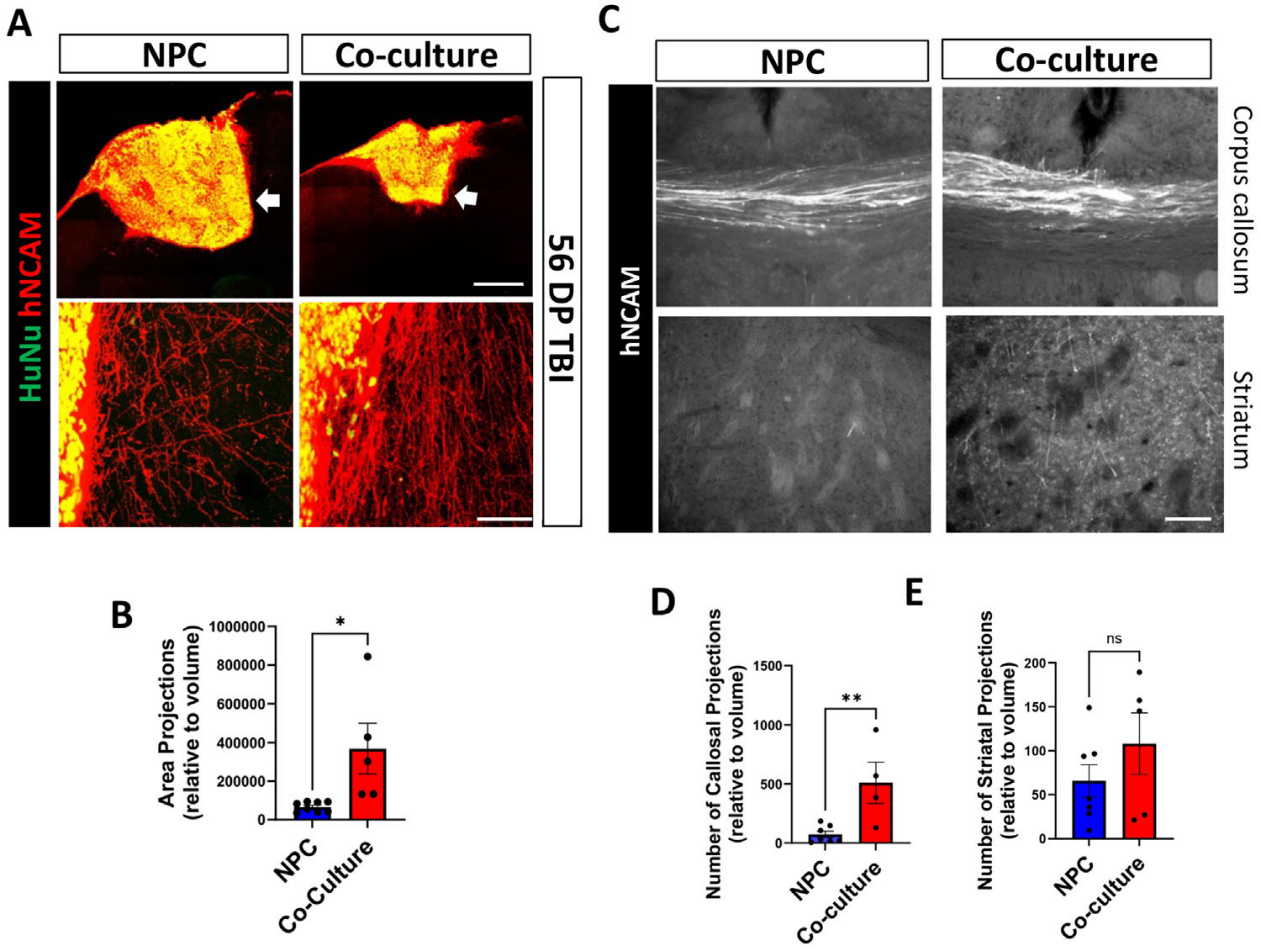
Increased relative graft‐host integration upon co‐culture implantation. (A) Representative confocal images of implanted NPC and co‐culture constructs at 56 DP TBI labelled with the human markers HuNu and hNCAM. Lower panels represent higher magnification images of the area indicated with the white arrows in the upper panels. Scale bars=500 µm (upper) and 50 µm (lower panels). (B) Quantification of hNCAM+ area adjacent to the implant normalized to the graft volume. Statistical significance was assessed using an unpaired t‐test (p < 0.05). (C) Representative Neurolucida images of axons immunolabelled with hNCAM detected in the corpus callosum and in the striatum. Scale bar=100 µm. (D) Quantification of number of axons detected in the corpus callosum normalized to the graft volume. Statistical significance was assessed using an unpaired t‐test (p < 0.01). (E) Quantification of number of axons detected in the striatum normalized to the graft volume. Statistical significance was assessed using an unpaired t‐test (ns=non‐significant).

Anatomical integration of implants with host tissue is crucial for tissue healing and functional recovery. To assess implant integration at 56DP TBI, we measured hNCAM+ area of processes extending outward from the implanted constructs (Figure 7A). Our analysis revealed that the co‐culture group had a relatively larger hNCAM+ area in the adjacent cortex, suggesting a higher degree of axonal growth into the host tissue (Figure 7B). We hypothesized that this difference could be due to changes in astrocytic scarring around the implanted constructs at this time point between the groups. However, we did not see a difference in GFAP border formation around the implants between the two groups (Figure S6A,B,C). To assess the capacity of implants to send out axonal projections to distant targets, we quantified axonal projections in the corpus callosum and striatum (Figure 7C). The co‐culture group sent out a higher number of projections per implant volume into the corpus callosum in comparison to the NPC only group (Figure 7D). The relative number of axonal projections in the striatum was not statistically different between NPC and co‐cultures (Figure 7E).

This data shows an increased relative number of axonal projections expanding from co‐culture implants when compared with NPC. We next asked if these axons were functionally integrated with the host.

### 2.7 Implanted human neurons present pre‐ and post‐ synaptic markers in the host cortex

**FIGURE 8:**
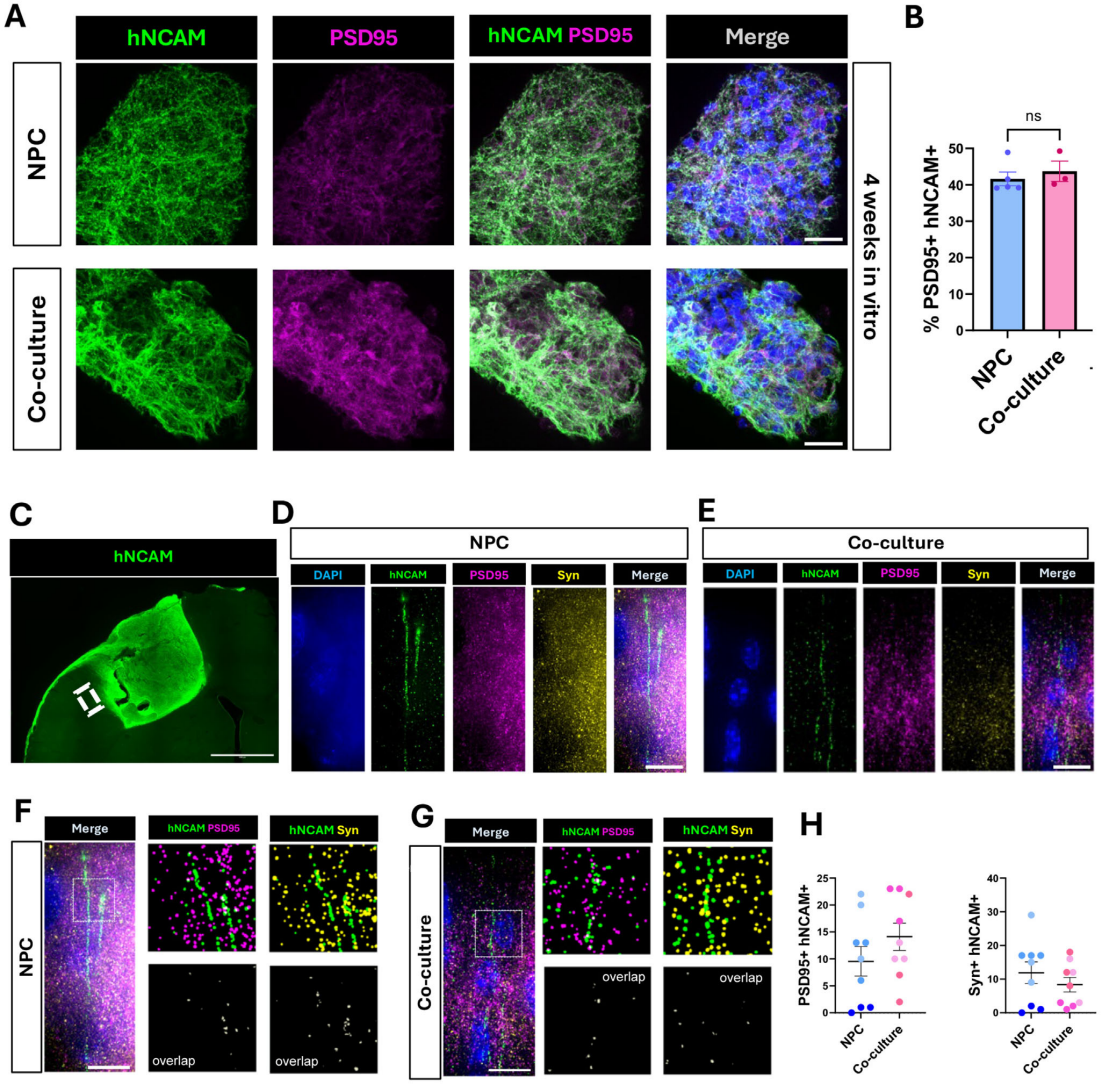
Synapse formation between implanted hNPCs and host murine neurons. (A) NPC and co‐culture 3D constructs in vitro at 4 weeks post microfluidics generation. Colocalization of human neuronal cell adhesion marker (hNCAM) and post‐synaptic density protein 95 (PSD95) suggests the formation of synapses within the constructs. Scale bar=20 µm. (B) The percent of PSD95+hNCAM+ was not significantly different between NPCs and co‐cultures (ns=non‐significant). (C) Overview tile of in vivo hNCAM labeling the construct and axons extending from the construct into the mouse cortex at two months post‐implantation. White rectangular box represents example of area imaged at high magnification within the adjacent mouse cortex. Scale bar=500 µm (D) Immunofluorescence of hNCAM, PSD95, and Syn in NPCs. Scale bar=10 µm (E) Immunofluorescence of hNCAM, PSD95, and Syn in co‐cultures. Scale bar=10 µm (F) Immunofluorescence of hNCAM, PSD95, and Syn in NPCs. Co‐localization in NPCs was evaluated using the synapse counter plug‐in available for Fiji. 20 µm segments of hNCAM were selected for co‐localization analysis. Scale bar=10 µm (G) Immunofluorescence of hNCAM, PSD95, and Syn in co‐cultures. Co‐localization in co‐culture was evaluated using the synapse counter plug‐in available for Fiji. 20 µm segments of hNCAM were selected for co‐localization analysis. Scale bar=10 µm (H) Percent overlap of both PSD95+hNCAM+ as well as Syn+hNCAM+ was similar between the NPC and co‐culture implants, as evaluated using an un‐paired t‐test.

One of the hallmarks of the microfluidics‐generated 3D constructs was that NPCs matured into process bearing neurons suggestng they may also express synaptic proteins. We asked if NPC or co‐cultures kept in vitro for 4 weeks expressed synaptic markers. We found a substantial overlap between hNCAM+ processes and immunofluorescence of the postsynaptic marker post‐synaptic density protein 95 (PSD95) (Figure 8A). Quantification of PSD95 puncta on hNCAM processes did not show differences between NPC and co‐cultures (Figure 8B).

We next asked if the hNCAM+ axons emanating from implants into the adjacent cortex were co‐expressing pre‐ and post‐synaptic proteins (Figure 8C). We used PSD95 and synaptophysin (Syn) to mark post‐synaptic and pre‐synaptic terminals, respectively (Figure 8D, E). Images were then segmented and colocalization was scored as hNCAM signal overlapping with PSD95 or Syn (Figure 8F, G). Quantification indicated that synaptic protein colocalization was similar between NPCs and co‐cultures (Figure 8H). Overlap between hNCAM and PSD95 suggests that the axons extending from the implant into the host are receiving synapses marked by the presence of this post synaptic marker on the axon. Similarly, co‐localization between hNCAM and the pre‐synaptic protein Syn suggests that the implant derived axons are forming synapses in the host cortex.

### 2.8 Implanted human neurons are functionally integrated to the host brain

**FIGURE 9:**
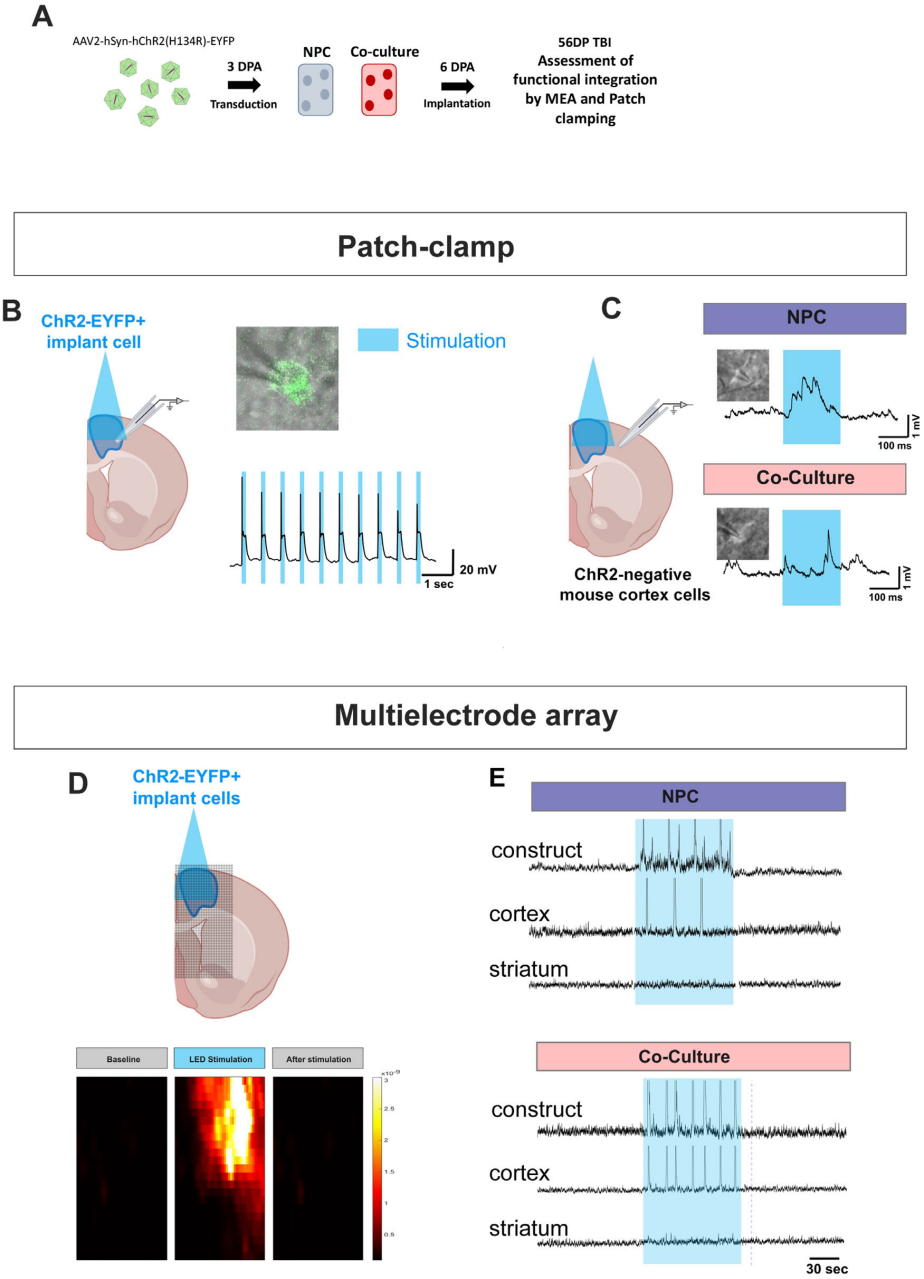
Electrophysiological integration of implants with host brains. (A) Schematic of the experimental timeline. (B) The left panel shows a schematic of a brain slice hemi‐section with ChR2‐EYFP labelling and cell stimulation with blue light in the implant 1‐month post‐implantation. The right panel shows a representative example of the evoked spiking events after optogenetic manipulation. Implant neurons can be targeted based on their expression of EYFP. Stimulation with blue light of ChR2‐EYFP labelled cell in the implant. (C) Recording from ChR2‐negative cells (n = 3‐4 neurons per group) in the adjacent cortex exhibited slow but detectable electrophysiological responses in both the NPC and co‐culture implants. Optogenetic activation of implant neurons induces depolarization on host‐ChR2‐EYFP negative neurons, indicating functional connectivity between neurons in the implant, and neurons in the host cortex. (D) Multielectrode array stimulation of implant results in robust responses in the implants of brain slice hemisections. Two months after implant, ChR2‐EYFP labelled implants were stimulated with blue light. Representative position of the array is shown on top of the brain slice. (E) Optogenetic stimulation with blue light shone on ChR2‐EYFP cells in the implant caused electrophysiological responses in the nearby cortex in NPC and co‐culture implants. Optogenetic stimulation induced evoked responses on electrodes in the surrounding areas (n = 3 slices per animal, 5 animals total). Example electrodes from inside the implant (top), on the surrounding area, 350 µm from the edge of the implant (middle) and at a distance of 1750 µm from the implant (bottom). Evoked activity was consistently recorded up to 700 µm from the edge of the implant.

To assess whether the implants functionally integrated with the host brain, we first transduced microfluidic‐generated 3D constructs in vitro with an adeno‐associated virus (AAV) vector encoding Channelrhodopsin‐2 (ChR2) fused to an enhanced fluorescent reporter (AAV2‐hSyn‐hChR2(H134R)‐EYFP) prior to implantation. ChR2 is a light‐gated ion channel that opens when stimulated with blue light, permitting entry of ions into the cell, which causes neuronal depolarization [60]. This allows for rapid and specific optogenetic modulation of the excitatory state of the implanted human cells, while identifying synaptic connections formed between the human and host murine neurons. Implant neurons were transduced to express Chr2‐EYFP so they could be optogenetically activated by exposing them to 405 nm blue light (Figure 9A). We confirmed transduction with fluorescence microscopy and visualized EYFP+ neurons at 6 DPA in vitro (Figure S7B).

byTo confirm functional activity of the Chr2‐transfected in the implant neurons, patch‐clamp recordings were done in EYFP‐positive neurons. These showed robust and immediate activation by light stimulation (Figure 9B). We then recorded the activity evoked by ChR2‐EYFP implant neurons of EYFP negative mouse neurons in the host surrounding cortical regions (Figure 9C). We were also able to evoke electrophysiological responses in Chr2‐negative host neurons through the activation of Chr2+ neurons in the implant (Figure 9C). This indicates that neurons in the mouse cerebral cortex received functional inputs from neurons in the implant.

Then, we evaluated the integration of the implant into the overall cortical circuitry with multi‐electrode array (MEA) recordings of the motor cortex and surrounding regions (Figure 9D). Optogenetic activation of the implant was sufficient to elicit brain‐wave activation of neuronal populations up to 700 µm away from the limits of the implant, in motor, prefrontal and somatosensory cortices (Figure 9E). Both approaches show evidence of functional integration of implanted neurons into the microcircuitry of the surrounding cortex in mice.

## 3 Discussion

This study demonstrates that murine astrocytes exert multiple beneficial effects on human neural progenitor cells, in vivo and in vitro, collectively leading to improved outcomes following brain implantation. Using live/dead assays, we found that the presence of astrocytes in co‐cultures significantly enhanced both in vitro and in vivo cell survival. Additionally, astrocytes boosted neuronal maturation, as indicated by increased expression of the pan‐neuronal marker NeuN and layer‐specific transcription factors. Importantly, we observed that constructs generated with microfluidics techniques survived implantation as effectively in vivo as did conventional cell suspensions. Upon implantation, astrocytes added to microfluidics‐generated constructs facilitated healing of the brain injury and increased axonal projections from the graft to the adjacent host cerebral cortex and corpus callosum. Co‐cultures also increased the association of astrocytic processes with BVs as well as the association of the water channel AQP4 with BVs in the implant. Thus, it is likely that astrocytes increased functionality of BVs inside the implants.

With our microfluidics approach to generating 3D constructs, we were able to scale up production of implants. It is possible to easily generate large numbers of microfluidics‐based constructs, a feature that could be especially important for drug screening [75]. Another definitive advantage of microfluidics approaches compared with other sources of implants that have been used, such as organoids, is that the former permits tight control of starting cell types, cell densities and extracellular matrix [74]. In contrast, organoids are notoriously variable, taking on multiple identities, shape and sizes within fields of undifferentiated and variable tissue. Thus, microfluidics‐generated constructs are preferable in terms of their physiological relevance. Importantly, we observed that constructs generated via microfluidics techniques survived implantation as effectively in vivo as did conventional cell suspensions. Given that high fluid pressure during microfluidics processing can compromise cell viability [60], this finding supports the feasibility of microfluidics approaches for neural implantation.

Notably, astrocytes contributed to a significant reduction in TBI lesion size. Compared with NPC‐only implants, co‐culture implants resulted in smaller or even macroscopically undetectable lesions, likely due to improved integration of the astrocyte‐containing constructs into the injury site. Interestingly, despite identical initial cell numbers, astrocytes reduced the construct size and increased its density in vitro. This outcome persisted in vivo upon implantation. Cells in the developing human cerebral cortex have very little extracellular space between them; they are dense, and co‐cultures replicated this scenario. Astrocytes are known to express adhesion molecules, which may also have contributed to this result [37].

While some hNPC‐only implants failed to adhere to the lesion site and were lost, astrocyte‐containing constructs showed significantly improved retention, suggesting astrocytes enhance implant stability. At 14 days post‐TBI, few NPC constructs remained, likely due to insufficient time for adherence and subsequent loss during tissue processing. In contrast, co‐cultures were better retained, indicating that astrocytes improved graft integration. Implantation success also improved at 56 days post‐TBI, consistent with the literature finding better repair at longer survival times [54, 61, 62]. Although extended post‐implantation periods are often needed to assess functional integration, our findings highlight that astrocytes enhance early implant retention and stability.

For brain implants to be therapeutically effective, they must establish axonal connectivity with the host tissue. This involves extending axons from the implants into the host brain and receiving appropriate inputs. This scenario necessitates precise pathfinding and neurotransmitter‐specific synaptogenesis. Previous studies have documented successful axonal outgrowth and connectivity in both cell suspension and organoid implants [5, 7, 10, 17, 62, 63]. In our study, we observed robust axonal outgrowth from both NPC and co‐culture constructs; however, the presence of astrocytes enhanced early axonal extension near the implant site. We examined two key output targets of the somatosensory cortex: the contralateral cerebral cortex and the striatum. Significantly more human axons in the co‐culture implant were detected in the corpus callosum, the axonal route to the contralateral cortex. We also found a trend toward increased axonal projections into the striatum. Axonal growth is necessary but not sufficient for functional circuits. Synapses, composed of pre‐synaptic and post‐synaptic protein expression, are needed for circuit activity. We showed evidence for both and found a considerable overlap between them and hNCAM‐labelled processes, proving they came from the human implants. Patch clamping of non‐human cells after optogenetic stimulation suggested the presence of functional synaptic contacts between the human ChR2‐transduced cells in the implant and host murine neurons. Confirming this, qualitative analysis of MEA readings in ex vivo implanted brain slices of the implanted brains indicated functional integration of human cells with the murine host neuronal network. The axonal projection experiments along with the electrophysiology readouts indicate that the projections sent out from the implants are physiologically functional. Further studies are needed to better understand the effect of astrocytes on the anatomical and functional integration of human neurons with the host brain upon implantation.

A unique aspect of our study was the heterochronic nature of the co‐cultures, in which astrocytes, which typically develop later in vivo, were combined with NPCs, which emerge earlier. Early NPC cultures naturally contain very few astrocytes, which led us to consider delaying co‐culture formation until human‐induced pluripotent stem cell (hiPSC)‐derived astrocytes matured. However, to expedite the process, we employed primary astrocytes isolated from the murine cerebral cortex.

To understand how the astrocytes promote neuronal maturation and survival, we analyzed hNPCs treated with co‐culture conditioned medium. Even though at 2 weeks no significant difference was seen in terms of maturation, analysis of secreted factors in culture media showed a significant increase in the expression OPN and IGFBP‐2, which are related to brain development and plasticity [31]. IGFBP‐2 is abundant during brain development and is expressed in the cerebral cortex by neurons as well as by blood vessel cells and has insulin receptor‐dependent and ‐independent functions [28, 29, 30]. IGFBP‐2 is also important for memory, likely through increasing intrinsic neuronal excitability and also facilitating long term potentiation [28, 29, 30]. Further studies could determine with functional assays, how these molecules coordinate blood vessel functions in implants. There was no difference in the expression IGFBP‐9, a matricellular protein which is involved in brain development and disease [33, 34, 35] or upregulation of VEGF expression, a regulator of vasculogenesis and angiogenesis [32], in 2D co‐culture cell medium. Interestingly, upon implantation, we found that both NPC‐only and co‐culture implants show the same level of angiogenesis. The correlation of these findings indicates that the difference between the two groups could be based not in the formation of new BVs, but rather their functionality.

Rodent astrocytes can be readily cultured with human neurons and are known to promote neuronal maturation, making them advantageous for physiological assessment of human neurons. Many studies have employed cross‐species co‐culture models pairing human neurons with murine or rat astrocytes, consistently demonstrating enhanced neuronal maturation and improved electrophysiological properties [64, 65, 66]. As a proof of concept, and to identify any differences in the influence of human astrocytes in the maturation of hNPCs, we performed experiments with human astrocyte co‐cultures. Co‐cultures with human astrocytes showed increased MAP2 expression at two weeks, which was not seen in the murine astrocyte co‐culture. While this may indicate a delayed pro‐maturational effect by murine astrocytes in comparison with the human co‐culture, murine astrocytes were still able to support increased maturation, axonal projection and blood vessel coupling upon implantation, exemplifying the robustness of our system.

Astrocytes in the human brain are substantially larger and more structurally complex than those of other mammals and are thought to contribute to species‐specific cognitive functions [37, 38, 39, 40, 41, 42, 43]. Remarkably, studies have shown that human glial progenitors transplanted into mouse brains not only grow in size but also enhance cognition [11, 67, 68]. In our study, we observed a striking increase in both the size and complexity of astrocytes within both sets of implants. These were host‐derived astrocytes that migrated into NPC implants or both host and primary murine astrocytes in the co‐culture group. The morphological changes of murine astrocytes suggest that the implants produced cell‐surface or diffusible factors that promoted astrocyte hypertrophy, the identity of which remains unknown. To our knowledge, there are no reports in the literature showing mouse astrocytes taking on a human morphological phenotype. Whereas mouse reactive astrocytes enlarge under pathological conditions, this plasticity does not reflect adoption of human astrocyte architecture (e.g., 2–3 times larger domains or 10 times more primary branches). Further experiments could clarify the molecular mechanisms causing astrocyte morphological changes and define the extent to which the human grafts reprogram murine glial identity. Single‐cell RNA sequencing of implanted murine astrocytes could determine whether these morphologically remodeled cells acquire a transcriptomic state that converges toward a human astrocyte–like signature. In addition, interrogation of signaling pathways and secreted factors associated with astrocytic hypertrophy, increased complexity, and functional maturation could provide mechanistic insight into the changes that astrocytes undergo upon implantation.

One of the rate‐limiting factors in long‐term survival and function of cortical implants is the lack of sufficient vascularization. Reactive astrocytes are known to upregulate pro‐angiogenesis genes, promoting vascular stabilization and interaction with endothelial cells. We identified blood vessels within both NPC and co‐culture implants. Given that astrocytes contribute to BBB formation and enhance endothelial function, we investigated their role in vascular integration. While astrocytes did not increase blood vessel density within the implants, they facilitated GFAP+ and AQP4+ physical associations with the vasculature, suggesting a role in BBB re‐establishment. AQP4, a key water channel involved in brain fluid homeostasis, is initially distributed throughout the astrocytic cell membrane but becomes progressively localized to endfeet as astrocytes mature [69]. This redistribution is essential for BBB function and cytotoxic oedema regulation following TBI. Our results suggested that the addition of astrocytes in the co‐cultures facilitated this essential function.

We further examined this question by studying the expression of β‐dystroglycan which is found at the gliovascular interface, bridging the extracellular matrix and the cytoskeleton [57, 58, 59]. It serves to anchor astrocytic endfeet to the parenchymal basement membrane by binding to laminin. It also helps link intracellular proteins like dystrophin and AQP4 [57, 58, 59]. We found β ‐dystroglycan expression in implant blood vessels that appeared normal. Additionally, we found abnormal expression, “flags” of this protein in cellular structures that were not polarized towards endfeet. Previously, β‐dystroglycan had been found in perivascular cells lacking endfeet, and which preceded endfeet formation in developing rat brain [70]. There were fewer “flag”‐like structures in the co‐culture implants than in the NPC constructs. Taken together, these data suggests that the addition of astrocytes accelerated endfeet polarization when compared to NPC constructs. Importantly, in both groups the distribution of dextran was similar and there was no evident sign of leakiness, suggesting a functional vasculature that is integrated with the host.

It is likely that the differentiation and maturation stages of cells used in our experiments affected the outcomes. We cultured NPCs during developmental periods when deep layer (early NPCs) and upper layer neurons (late NPCs) are being generated [36, 44]. Additionally, cells likely corresponding to ventricular zone (VZ) stem cells, as well as young post‐mitotic neurons could be found in the constructs. It may be that hESCs cultured at earlier stages and thus with a greater ratio of VZ stem cells to downstream daughter cells would not have responded as favorably to the inclusion of astrocytes as in this study. Conversely, it may also be that co‐culture at later stages of neuronal differentiation would represent a more realistic embryonic scenario since this is when astrocytes appear. This latter approach may result in astrocytes having even greater beneficial effects.

The age of the host brain at the time of implantation was another critical factor. We implanted constructs into 7‐9 day old (P7‐9) mice, a stage chosen due to its high neuroplasticity, which facilitates synaptic integration and functional recovery. This developmental window is particularly relevant for neonatal injuries, including TBI, cerebral palsy, and perinatal hypoxia‐ischemia, where stem cell therapies have shown promise [71]. Stem cell therapies at these early stages of life are likely to be beneficial [72]. However, the effectiveness of astrocyte co‐cultures at later developmental stages or in adult brains remains uncertain. It would be informative to query whether adult astrocytes could be equivalently beneficial in implants. scRNAseq analyses have pointed to transcriptome heterogeneity in astrocytes throughout development and when they become reactive [73]. Another important consideration is the relatively beneficial effects of stem cell‐like astrocytes compared with resting adult parenchymal astrocytes and reactive adult astrocytes [73]. This is especially important to consider as TBI causes astrocyte reactivity, which will likely have profound effects on implant survival [1]. There will likely be ideal astrocyte stages or types to use in the regeneration of various types of brain injuries.

TBI in infants and children is an important cause of disability and mortality [74]. It can be caused by several events such as head injury during delivery, accidents, or abuse [75]. The young brain and skull possess unique biomechanical characteristics, such as increased plasticity and a more malleable skull, which influence their response to injury. This raises important questions about how implants might affect motor and cognitive recovery in this age category [74]. The postnatal brain is much more plastic and able to resolve injuries than the adult brain. However, we believe that older brains could also benefit from cell implantations. Work from the Gaillard and Vanderhagen labs, as well as others, demonstrates that the adult rodent brain can support stem cell implant‐derived axonal outgrowth, synaptogenesis, and behavioral recovery after TBI [5, 7, 8, 10]. Future work should explore whether our proposed strategy is also relevant in adult TBI models. Testing the effects of human iPSC‐derived astrocytes in this context, together with behavioral assays to demonstrate functional recovery, would represent an important step toward clinical translation.

Another concern in implantation work is the role of immunogenicity and implant survival. Immune reactions and graft‐versus‐host disease remain major obstacles in regenerative medicine, particularly with allogeneic transplants between genetically distinct individuals. Autologous implants avoid many of these issues but are not always feasible. In this study, we employed xenografts of human neural progenitor cells implanted into murine hosts. Because xenografts normally provoke immune rejection, we used NOD‐SCID gamma (NSG) mice, an extensively characterized strain routinely chosen for human cell engraftment, to ensure robust survival and integration of the implanted human cells without the need for additional immunosuppression.

There are several other gaps in our work that emerged and will guide future work. We do not know the long‐term effects of astrocyte co‐culture. For example, it will be fascinating to determine if murine astrocytes stay larger or if they regress to normal mouse astrocyte size and complexity. We also do not know if the morphological changes that occurred would result in functional changes in astrocytes. Human astrocytes implanted into rodent brain have been shown to improve behavioral tests [67]. Ultimately, it will be important to demonstrate improved behavioral recovery for the translational relevance of our paper. We are now poised to assess if the implanted or host murine astrocytes that immigrate into human NPC constructs can improve appropriate behaviors. Also, as mentioned above, it will be useful to know if longer‐term survival of co‐cultures increases site‐appropriate electrophysiological activity and function. Finally, it is likely that other brain TBI locations such as in the entorhinal cortex and visual cortex (memory and visual functions, respectively) will positively respond to inclusion of astrocytes—this needs to be tested.

## 4 Conclusion

This study provides evidence that astrocytes incorporated into microfluidics‐generated cortical constructs enhance neuronal survival, maturation, axon outgrowth, and vascular integration following TBI in a murine model. Future work should further evaluate the optimal astrocyte origin and timing leading to the best therapeutic outcome. In the long term, the integration of astrocytes into engineered neural implants may bring us closer to restoring neural function and mitigating behavioral deficits following brain injury.

## 5 Materials and Methods

### 5.1 Murine Astrocyte Isolation and Culture

Cortical astrocytes were isolated from postnatal day 1 (P1) C57BL/6 pups using a modified protocol based on [76]. Following terminal anesthesia and death by cervical dislocation, the brain was removed from the skull. The cerebral cortex was isolated and transferred to microtubes containing 1 mL of 1X HBSS (Life Technologies). After mechanical trituration, the tissue was incubated in 0.25% trypsin (Life Technologies) for 15 min. Trypsin was neutralized by the addition of the same volume of fetal bovine serum (FBS) (Life Technologies), and the resulting homogenate was centrifuged at 0.2G for 5 min. The supernatant was discarded, and the pellet was resuspended in DMEM F12 (Life Technologies) supplemented with 10% FBS, 2% Glutamax (Life Technologies) and 1% Penicillin/Streptomycin (Gibco). Cells were then plated onto T25 flasks, and the medium was changed every two days. Viability was determined with trypan blue and only healthy cultures (>90% survival) were plated.

Upon reaching 80% confluence, cells were passaged. Before passaging, flasks were kept in an orbital agitator for about 2 h at 180 rpm for removal of microglia at RT. The medium was then removed, and cells were washed once with sterile D‐PBS (Merck). 0.25% trypsin was then added, and cells were incubated for 5 min at 37°C. Trypsin was neutralized with an equal volume of FBS, and cells were collected in a 15 mL centrifuge tube for centrifugation at 0.3G for 5 min. The supernatant was discarded, and the pellet was resuspended in supplemented DMEM‐F12 previously mentioned and cells were plated onto new T25 flasks, at a ratio of 1:2. Astrocytes were used between the first and third passage for all experiments.

### 5.2 Neural Differentiation of human Embryonic Stem Cells

Neural induction of embryonic stem cells (line H9) was performed based on the protocol established by [36]. H9 cells (gift from Eric O'Neill) were thawed and plated onto 100mm^2^ plates coated with Geltrex (Gibco) and cultured in mTser Plus medium (StemCell Technologies). Upon thawing and passaging cells, the culture medium was supplemented with 0.1% 10 mM Y‐27632 dihydrochloride, a ROCK inhibitor (Stratech Scientific). The medium was changed every day.

When 80% confluent, cells were passaged at a 1:5 ratio, where 4 parts were plated onto two Geltrex‐coated wells from a 6‐well plate. For this, cells were incubated with 0.5 mM EDTA (Gibco) for 5 min at room temperature. Cells were lifted using a cell scraper and plated onto the wells. When cells reached 100% confluence, usually the next day, medium was changed to Neural Induction Medium (NIM), consisting of Neural Maintenance Medium (NMM) [50% DMEM‐F12, 50% Neurobasal medium (Life Technologies), 1% Glutamax, 1% B27 50X (Life Technologies), 0.5% N2 100x (Life Technologies)] supplemented with two SMAD inhibitors, 1 mM LDN193189 (Sigma) (1:1,000) and 1 mM SB431542 (Tocris) (1:10,000). Neural induction was carried out for 7 days, with daily medium changes. On day 7, cells were passaged as previously mentioned, at a ratio of 2:3 onto poly‐L‐ornithine (PLO) (Sigma) and laminin (Sigma)‐coated wells and maintained in NIM with ROCK inhibitor. The medium was changed the next day to NMM only. The medium was changed every day. On day 12 of neural induction, cells were passaged in the same way as day 8. On day 19, passaging was done using StemPro Accutase (ThermoFisher) with incubation at 37°C for 5 min. Cells were collected in a centrifuge tube containing DMEM‐F12 and centrifuged at 0.2G for 5 min. Cells were plated onto PLO and laminin‐coated wells in NMM medium with ROCK inhibitor added. New passages were done every four days or when cells reached 80% confluency, following the 3:5 ratio. NPCs at day 31‐56 were used for experiments.

### 5.3 Murine Astrocyte Co‐Culture and Conditioned Medium Treatment

Cells were plated at a density of 120,000 cells per cm^2^ on PLO and laminin‐coated µ‐Slide 18 well plates (IBIDI, Thistle). For NPC/astrocyte co‐cultures, a ratio of 1:3 (murine astrocyte:NPCs) was used.

For conditioned medium experiments, cells were seeded as previously mentioned. Two groups were established, NPC‐only treated with NMM and NPC‐only treated with conditioned medium. Complete medium changes were performed every other day and, on those days, medium from murine co‐cultures maintained in 6 well plates was transferred to the conditioned medium group in the IBIDIs. The other group received NMM. This was done for a period of 2 weeks, when all groups were collected and fixed with 4% paraformaldehyde.

### 5.4 Human Astrocyte Co‐Culture and Collection

Fetal human cortical astrocytes (ScienCell, donor #38223) were cultured with NPCs up to passage 2 under conditions similar to those used for the murine co‐cultures. Cells were seeded at a density of 120,000 cells/cm^2^ on PLO‐ and laminin‐coated µ‐Slide 18 Well plates (IBIDI, Thistle Scientific). For NPC/astrocyte co‐cultures, a ratio of 1:3 human astrocyte to NPC was maintained. Collection of samples was done after 2 weeks and fixed with 4% paraformaldehyde for immunofluorescence.

### 5.5 Immunocytochemistry of 2D Cell Culture

Cells were plated at a density of 120,000 cells per cm^2^ on PLO and laminin‐coated µ‐Slide 18 Well plates (IBIDI, Thistle). For NPC/astrocyte co‐cultures, a ratio of 3:1 (NPC:murine astrocytes) was used. After 2 or 4 weeks, cells were fixed with 4% paraformaldehyde for 15 min and washed three times for 10 min with 0.1 m PBS. Samples were permeabilized with 0.5% Triton X‐100 in PBS for 15 min and blocked with 5% bovine serum albumin (BSA), 0.2% Triton X‐100 in PBS for 1hr. Then, cells were incubated with primary antibodies (Table 1) diluted in blocking solution overnight at 4°C. After three washes with 0.1 M PBS for 10 min, cells were incubated in secondary antibody (Table 2) solution containing DAPI for 1hr at room temperature and then washed three times for 10 min with 0.1 m Phosphate Buffer. The wells were filled with Fluorsave reagent (ThermoFisher) to preserve fluorescence in storage. Plates were stored at 4°C until imaged.

**TABLE 1:** Primary antibodies.

Antibody	Target	Dilution	CAT	Company
Mouse α‐TUJ1	β‐tubulin 1	1:200	ab78078	Abcam, UK
Chicken α‐MAP2	Microtubule‐associated protein	1:200	ab5392	Abcam, UK
Rat α‐GFAP	Glial fibrillary acidic protein	1:200	13‐0300	Thermo Fisher, USA
Rat α‐CTIP2	CTIP2 transcriptional factor	1:200	ab18465	Abcam, UK
Rabbit α‐SATB2	SATB2 transcriptional factor	1:200	ab92446	Abcam, UK
Rabbit α‐Nestin	Cytoplasmic Nestin protein	1:200	sc23927	Santa Cruz, USA
Rabbit α‐Cas3	Cleaved Caspase‐3	1:200	9661s	Cell Signalling, UK
Mouse α‐PHi3	Phosphorylated H3 histidine	1:200	06‐570	Millipore, USA
Rat α‐Ki67	Mitosis‐associated protein	1:200	14‐5698‐82	Invitrogen, USA
Mouse α‐S100β	Cytoplasmic astrocyte marker	1:200	S2532	Millipore, USA
Mouse α‐HuNu	Human nuclear antigen	1:200	MAB1281	Merck, USA
Rabbit α‐hNCAM	Human neural cell adhesion protein	1:200	ab75813	Abcam, UK
Rabbit α‐AQP4	Aquaporin‐4	1:100	ab128906	Abcam, UK
Mouse α‐βDG	β‐dystroglycan	1:200	sc‐33702	Santa Cruz, USA
Guinea pig anti‐PSD95	PDZ domain of mouse PSD95	1:200	124 308	Synaptic Systems, Germany
Mouse anti‐Synaptophysin	synaptophysin, presynaptic vesicle protein	1:200	14‐6525‐82	Invitrogen, USA

**TABLE 2:** Secondary antibodies.

Antibody	Target	Dilution	CAT	Company
AlexaFluor 488	α‐rabbit	1:500	A21441	Invitrogen, USA
AlexaFluor 594	α‐mouse	1:500	A21203	Invitrogen, USA
AlexaFluor 647	α‐rat	1:500	A21449	Invitrogen, USA
AlexaFluor 488	α‐rabbit	1:500	A11034	Invitrogen, USA
AlexaFluor 568	α‐mouse	1:500	A10037	Invitrogen, USA
AlexaFluor 647	α‐rat	1:500	A21247	Invitrogen, USA
AlexaFluor 488	α‐chicken	1:500	A11039	Invitrogen, USA
AlexaFluor 568	α‐guinea pig	1:500	A11075	Invitrogen, USA

### 5.6 RNA Extraction From Cell Cultures and qPCR Analysis of Target Genes

Cells were plated in 6 well plates at the same density and ratio as previously mentioned. At the 2 weeks timepoint, cells were lifted with StemPro Accutase (Gibco), and RNA was extracted from the cell pellet with the RNeasy Micro Kit (Qiagen) according to the manufacturer instructions. Total RNA was thawed on ice and quantified prior to reverse transcription. For each sample, RNA was combined with random primers and dNTPs, heated at 65°C for 5 min, and chilled on ice. Reverse transcription was performed using SSIII RT, RNaseOUT inhibitor, DTT, and 5x First‐Strand Buffer under the following conditions: 25°C for 5 min, 55°C for 1 h, and 70°C for 15 min. The resulting cDNA was diluted with RNase‐free water and stored at −20°C. Quantitative PCR was conducted using SYBR Green Master Mix with primers for BRN2, CTIP2, CUX1 and PAX6 (Table 3) and cDNA template. Reactions were run in technical quadruplicate under a standard melt‐curve protocol, and data were analyzed using GraphPad Prism 10.

**TABLE 3:** PCR primers.

PRIMER	Expression	Sequence	Citation
PAX6	Primary progenitor cells	F GCCAGCAACACACCTAGTCA R TGTGAGGGCTGTGTCTGTTC	[77]
CTIP2	Deep‐layer neurons	F GAGTACTGCGGCAAGGTGTT R TAGTTGCACAGCTCGCACTT	[77]
BRN2	Progenitor cells and upper‐layer neurons	F GACCTTTGCAGGCGAGTAAC R TCAGGAAGCTGCATTTTGTG	[77]
CUX1	Progenitor cells and upper‐layer neurons	F GCTCTCATCGGCCAATCACT R TCTATGGCCTGCTCCACGT	[77]
GAPDH	Housekeeping	F ACATCGCTCAGACACCATG R TGTAGTTGAGGTCAAGGG	IDT, USA

### 5.7 Proteomic Profiling of Angiogenic Soluble Factors in Cell Media

The relative expression levels of 31 proteins associated with mouse angiogenesis in cell culture supernatants were detected using a Proteome Profiler Mouse Angiogenesis Array Kit (R&D Systems). At the 2 weeks timepoint, medium from the 6 well plates from both NPC‐only and murine co‐cultures was collected, centrifuged and processed according to the manufacturer instructions. Four biological repetitions were carried out for this experiment. Pixel densities on the membranes were analyzed using FIJI. An average of 2 dots per antibody was calculated for each membrane, which was normalized by the positive control. The fold change compared to NPC was calculated.

### 5.8 Microfluidics‐Based Generation of 3D Constructs

#### 5.8.1 Fabrication of the PDMS chip

A pre‐printed master mould featuring a T‐shaped junction and connecting tubes was used. It was placed in a Petri dish, and a gentle blow with compressed nitrogen was used to clean it. A Sylgard 184 Silicone Elastomer Kit was used to prepare the Polydimethylsiloxane (PDMS) for the fabrication of T‐junction from the master mold. A total of 55 grams was weighed, consisting of 5 g of the curing agent and 50 g of the polymer base, maintaining a ratio of 1:10 PDMS. The pre‐cured PDMS was mixed thoroughly to ensure the curing agent was evenly distributed before being poured into the master mold. Degassing the mixture was achieved by placing the mixed pre‐cured PDMS in a vacuum desiccator and evacuating the chamber for a minimum of 30 min. During degassing, bubbles appeared and raised to the surface of the mixture and popped. The chamber was vented as bubbles came close to the surface; this was repeated 2–3 times for the first 15 min. Degassing was considered complete when no bubbles are visible in the mixture.

For curing, the Petri dish was covered and placed in an oven heated to 65–80°C for an hour. PDMS casting was then removed from the oven and placed on a clean bench top. A scalpel was used to make straight cuts along the edge of the master mould to detach the cured PDMS. Once all the edges were released, the PDMS cast was gently lifted from the bottom of the mould and placed in a clean Petri dish. Two identical PDMS casts were perfectly aligned side by side to form a complete set of a microfluidics T‐junction chip.

#### 5.8.2 Bioink Preparation and Fabrication of the 3D‐Neuronal Constructs

A monolayer of hNPCs with or without murine astrocytes were harvested on day 30–45 of differentiation. Initially, the cells were washed with DPBS twice, followed by a 5 min incubation with 1 mL of pre‐warmed Accutase. After that, 5 mL of neural maintenance media (NMM) was added to each well for resuspension of the cells and transferred to a 15 mL Falcon tube. To pellet the cells, centrifugation was performed at 1200 rpm for 5mins, followed by discarding the supernatant. In certain instances, an additional 1 min centrifugation was employed to ensure complete removal of the supernatant.

Cell density for all groups was 8X10^7^ cells/mL. NPCs were isolated between day 31–56 of neural induction. For the co‐culture group, murine astrocytes and NPCs were lifted at the same time, mixed at a ratio of 3:1 (NPC:murine astrocytes) and centrifuged. The pellet was then resuspended in pre‐thawed Matrigel. The constructs were generated using the fabricated microfluidics chip connected with a three‐way tubing system, two inlets and one outlet. One inlet tubing was loaded with bio‐ink, cells resuspended in Matrigel (Corning), the other inlet was loaded with undecane oil (Merck), and the outlet tubing collected cell‐laden Matrigel droplets. The flow rate of oil and bio‐ink and the diameter of collection tubing defined the dimensions of the constructs. Once the outlet tube was filled with droplets, it was sealed and incubated at 37°C for 2hr to facilitate the gelation of Matrigel. Following gelation, the cell‐laden Matrigel droplets were collected and transferred into low‐attachment 24‐well plates for further in vitro culture.

### 5.9 LiveDead Assay for 3D Microfluidics‐Based Constructs

Cell viability was assessed by using calcein‐AM (Cambridge Biosciences Ltd). Calcein AM is a non‐fluorescent, cell‐permeable molecule that is cleaved by intracellular esterases within the cell, producing green fluorescence in live cells. Propidium iodide (Sigma Aldrich), in turn, permeates the membranes of dead cells, binding to their DNA and emitting red fluorescence. Microfluidics‐based 3D constructs were incubated with calcein‐AM and propidium iodide at 37°C and 5% CO_2_ for 20 min. Images were acquired using a confocal microscope with a 20X air objective. The constructs were imaged in a z‐stack with 5 µm intervals, capturing at least five to six optical planes per construct to encompass the entire structure.

### 5.10 AAV‐Mediated Channelrhodopsin Transduction of 3D Neural Constructs

3D microfluidic constructs containing either hESC‐derived NPCs alone or co‐cultures with primary murine astrocytes were transduced with channelrhodopsin to enable optogenetics electrophysiological studies of functional integration of implants with the host brain. For this, 3D cell constructs were produced by the microfluidic‐based technique previously described. Following 3 days of maturation in NMM, constructs were transduced with an adeno‐associated virus (AAV) vector. pAAV‐hSyn‐hChR2(H134R)‐EYFP was a gift from Karl Deisseroth (Addgene plasmid # 26973; http://n2t.net/addgene:26973; RRID:Addgene_26973). Viral particles were added directly to the culture medium at 200,000 viral particles per cell, and constructs were incubated under standard culture conditions (37°C, 5% CO_2_) for 24hr to allow for efficient transgene expression. Complete medium change was performed and 48 h later, implantation was done. Expression of ChR2 was confirmed in vitro by fluorescence microscopy in constructs kept in vitro for 5 days (Figure S7).

### 5.11 Mice

All animal experiments were approved by a local ethical review committee and conducted under the UK Animals (Scientific Procedures) Act, 1986 (ASPA), under valid personal and project license (PP3146325). Animals were bred and housed in individually ventilated cages (IVCs) on a 12‐hour light/dark cycle in the Biomedical Sciences Building, Oxford. Water and food were given ad libitum. Cages were environmentally enriched with an activity wheel, nesting material, nestlets, chew stick and forage mix. NOD‐SCID gamma (NSG) mice were acquired from Charles River. Postnatal animals at P7‐P9 were used for all in vivo experiments.

The NOD‐SCID gamma (NSG) mouse combines multiple immunodeficiency traits to create a highly permissive host for human cell engraftment. Its background is the NOD/ShiLtJ strain, which already harbors defects in innate immunity—such as compromised complement activity, impaired macrophage phagocytosis, and suboptimal dendritic cell function—as well as alterations in tolerance‐related pathways. On this foundation, the SCID mutation in the Prkdc gene abolishes functional V(D)J recombination, resulting in an absence of mature B and T lymphocytes. Further, deletion of the interleukin‐2 receptor common gamma chain (Il2rg) disrupts signaling through several cytokine receptors (including IL‐2, IL‐4, IL‐7, IL‐9, IL‐15, and IL‐21), eliminating natural killer (NK) cell development, and further weakening any residual adaptive or innate responses. As a result, NSG mice lack mature B cells (and thus immunoglobulins), T cells, NK cells, and have minimal antibody‐ or T cell–mediated immunity. This profound immunodeficiency allows robust engraftment of human hematopoietic stem and progenitor cells, reconstruction of human immune system components, and growth of human tumor xenografts with minimal rejection.

### 5.12 Intracerebral Cell Suspension Injection

Two groups of mice were generated based on the cell types injected: NPC only and 3:1 NPC:Astrocyte (co‐culture). NPCs were derived from H9 cells as previously described. From day 19 of the neural induction protocol, NPCs were either cultured alone or in the presence of primary murine astrocytes, until day 31‐56 of the neural induction protocol. On this day, cells were injected into the cerebral cortex. Briefly, 100,000 cells were resuspended in 1 mL of sterile PBS containing Fast Green (0.1%) (Merck) and collected in a Hamilton syringe coupled to a Stoelting injector. Postnatal mice (P7‐9) were anesthetized with isoflurane and placed in a custom support mould. 1 mL of cell solution was injected through the skin and skull, into the motor/somatosensory cerebral cortex. After anesthesia recovery, pups were returned to the dam and 2 weeks later, they were perfused with 0.1 M PBS followed by 4% paraformaldehyde after terminal anesthesia. In total, 7 animals across 3 litters were injected in each group.

### 5.13 3D construct Implantation Following TBI

As previously described, microfluidics‐based 3D constructs containing either NPCs or a co‐culture of NPCs and primary murine astrocytes were generated. These constructs were implanted into the motor/somatosensory cortex 3 to 7 days post‐assembly (DPA), immediately following traumatic brain injury (TBI). Postnatal mice (P7‐9) were anesthetized with isoflurane and secured in a stereotaxic apparatus. Following a skin incision and craniotomy, an aspiration lesion was performed in the motor/somatosensory cortex. The 3D constructs were then positioned within the lesion site, and the bone flap and skin were closed using surgical adhesive. For the TBI only group, the same procedure was followed without the implantation step. The animals were returned to the dam and, after a 14 day or 56 day recovery period, underwent perfusion fixation after terminal anesthesia. Constructs were implanted into a litter of 11 animals for the 14 day timepoint. For the 56 day timepoint, constructs generated from five litters were implanted in a total of 40 animals. Animals in which constructs were not found were excluded from further analysis. This happened at a higher frequency in the NPC group at the 14 day timepoint (5 out of 6 animals) than the co‐culture group (1 out of 5). At the 56 days timepoint, only 5 out of 21 animals from the NPC group, and 5 out of 12 animals from the co‐culture group lacked constructs.

### 5.14 Immunohistochemistry of Brain Sections and 3D‐printed Constructs

Brains from TBI alone, and implanted animals (both cell suspension injections and 3D construct implantation) were collected after perfusion with 4% paraformaldehyde and maintained in 30% sucrose solution for 24 h. The implanted brains were sectioned on a freezing microtome and the resulting sequential 30 micrometer thick sections were kept at 4°C. For immunohistochemistry of brain sections and whole 3D constructs, samples were washed three times in 0.1 M PBS and then blocked for 1hr in 10% donkey serum, 0.5% Triton X‐100 in PBS. Then, samples were transferred to primary antibody (Table 1) diluted in blocking solution and incubated overnight at 4°C. For immunohistochemistry of the synaptic markers synaptophysin and postsynaptic density protein 95 (PSD95), samples were incubated in primary antibody for two nights at 4°C. After three washes with 0.1 M PBS, samples were incubated in secondary antibody (Table 2) solution with DAPI, diluted in blocking solution, for 1 h at room temperature and washed in 0.1 M PB. Brain sections were then mounted onto microscope slides and 3D constructs were kept in 18‐well plates filled with Fluorsave (Merck) until imaged. Brain sections used for synaptic protein evaluation were coverslipped using ProLond Diamond Antifade Mountant (ThermoFischer). Immunohistochemistry was compared to no‐primary, secondary‐only controls to assess non‐specific binding.

### 5.15 In Vivo Injections of Fluorescein‐conjugated Dextran

Two months post‐implantation, mice were administered 0.1 mL of 4 mg/ml solution of fixable Fluorescein‐conjugated 70 KDa Dextran (ThermoFisher Scientifics) via tail vein injection. Animals were collected via overdose of anesthetic 30 min after dextran administration. Brains were then fixed for 24 h with 4% paraformaldehyde. After cryopreservation with 30% sucrose for 48 h, brains were frozen and free‐floating 30 µm thick sections were obtained with a Leica microtome.

### 5.16 Image Analysis

All images were taken either with a ZEISS LSM 710 confocal microscope using ZEN software, Leica epifluorescence microscope (DMR) equipped with a Leica DC500 CCD camera magnifier or a Nikon ECLIPSE Ti inverted microscope system using Volocity Version 7.0.0. Mouse brain tile scans were produced using the Nikon ECLIPSE Ti inverted microscope with a 10x objective with a 20% overlap and brightness but no shading. Images were analyzed using Fiji (NIH) or QuPath.

#### 5.16.1 Analysis of Immunocytochemistry—Nuclear Markers; CTIP2, SATB2, KI67, aCAS3, NeuN, PI

For quantification of nuclear markers, manual counting was performed using the cell counter tool in Fiji. Nuclei exhibiting overlap of the desired marker with DAPI or HuNu, a human‐specific nuclear marker were considered positive for analysis. In vivo quantification of SATB2, CTIP2, Ki67 and aCasp3 in implanted 3D constructs was performed using QuPath.

#### 5.16.2 Cytoplasmic Marker and Histological Analysis, MAP2, Nestin, Calcein AM

For quantification of cytoplasmic markers, a macro was written in Fiji. A threshold was set before the selection of area and measurement. All measurements were normalized by the total area of each image. For this, images were acquired on the Nikon ECLIPSE Ti inverted microscope with a 20x objective. For quantification of degenerating axons, the MAP2 marker was used to visualize axons. Individual axons presenting the morphological hallmark of Wallerian degeneration were counted.

#### 5.16.3 Lesion Area on Brain Surface

After collection, brain images were imported to Fiji. The visible lesion area on the brain surface was measured by drawing a freeform shape around the visible lesion.

#### 5.16.4 Analysis of Volume and Density of Constructs in Vitro and In Vivo

For analysis of the volume of 3D constructs in vitro, z‐stack images were acquired on a ZEISS LSM 710 confocal microscope. The Z‐stacks were delineated, and the volume was calculated using Neurolucida software. For density of 3D constructs in vitro, the total number of nuclei was counted using QuPath and the number divided by the volume. Volume measurements in vivo were calculated using Neurolucida. Grafts were delineated in serial coronal sections 240 µm apart.

#### 5.16.5 Axonal Projection Analysis

The analysis of hNCAM projection in the cortex adjacent to the implants was done using Fiji. Tile‐scans containing the graft were acquired on a ZEISS LSM 710 confocal microscope using a 20x objective. A Region of Interest (ROI) was delineated around the graft (HuNu+ region). A threshold for hNCAM was generated and the total area calculated, as well as the area inside the ROI. The projection area was the difference between total hNCAM area and hNCAM area inside the ROI. The average projection area of at least 3 coronal sections per animal was normalized by the volume of the construct. Sections stained for hNCAM and DAPI were used for implant axonal outgrowth into the corpus callosum or striatum. Images were acquired with a Leica epifluorescence microscope (DMR) equipped with a Leica DC500 CCD camera magnifier using a 20x objective. For the axonal outgrowth into the striatum analysis, the most anterior section which contained the implant was selected (section 1) and then the next four sections immediately posterior were used for quantification (five sections total per animal). In each section, a 20x image was acquired in the striatum ipsilateral to implant and directly below the implant site. For axonal outgrowth into the corpus callosum, section 1 was designated as the first section in which the corpus callosum appeared. This section and the next four sections posterior were used for quantification. Each image was acquired centered with the corpus callosum crossing between the two hemispheres. Images were opened in Fiji and axons with positive hNCAM staining in either the striatum or corpus callosum respectively were manually counted using the cell counter plugin in Fiji.

#### 5.16.6 Sholl Analysis

For Sholl analysis, images were acquired on a Zeiss LSM 710 confocal microscope with a 40X objective. 27 GFAP+ astrocytes from within the graft were traced across 5 animals per group using Neurolucida software. Only astrocytes fully contained within the grafts were included for analysis. From the traced cell skeletons, the total number of intersections and the area covered were extracted. To assess the complexity and branching pattern of astrocytic processes, Sholl analysis was performed by plotting the number of intersections as a function of radial distance from the cell soma. This method allows for the calculation of Sholl decay, which reflects the rate at which process branching decreases with increasing distance from the cell body, providing a quantitative measure of morphological complexity.

#### 5.16.7 Co‐Localization Analysis of (S100β/GFAP, Lectin/GFAP, Lectin/AQP4)

For co‐localization of S100β/GFAP, Z‐stacks were acquired using a 20x objective in a Nikon ECLIPSE Ti inverted microscope. Images were preprocessed with Gaussian filter (Sigma = 2) and background subtraction (Sigma = 50). JACoP BIOP Fiji plugin was used to calculate the Manders coefficients M1 and M2 in the region of interest (ROI). For co‐localization of lectin with GFAP or AQP4, Z‐stacks were acquired using a 40x objective on a ZEISS LSM 710 confocal microscope. A Macro was created in Fiji for generating an ROI around lectin+ areas. Inside the lectin ROI, a threshold for GFAP and AQP4 was generated, and the area calculated. Average percentage was calculated for 2‐3 images of a minimum of 2 brain sections per animal.

#### 5.16.8 Blood Vessel Length

To measure lectin‐labelled blood vessel length within implanted constructs, three images were captured per animal using a 10X objective and an EVOS FL Auto 2 microscope. This analysis included 8 animals in the NPC group and 5 in the co‐culture group. Only lectin+ blood vessels located within the grafts were included in the analysis. Vessel lengths were measured manually in Fiji using the freehand selection tool. The total vessel length (in mm^2^) was divided by the implant area, averaged per animal, and plotted.

#### 5.16.9 Analysis of S100β Density in the Construct

For quantification of the astrocyte marker S100β, manual counting was performed with the aid of the cell counter tool in Fiji. The area of HuNu+ cells was measured by using the ROI tool in FIJI to outline the area of HuNu positive cells, then applying the measurement tool in FIJI to calculate the area within the ROI. Most images were fully within the HuNu area, in which case the area of the image was used to measure the area of HuNu.

#### 5.16.10 Analysis of β‐Dystroglycan in the Constructs

For analysis of β‐dystroglycan, 5 NPC and 4 Co‐Culture implants were used. Z‐stacks were acquired using a 10x objective in a Nikon ECLIPSE Ti inverted microscope. A macro was written in Fiji wherein the background was removed and a gaussian filter (sigma = 4) was applied to images before measuring the area occupied by β‐dystroglycan. The percentage was obtained by dividing this value by implant total area, measured manually on Fiji. The average percentage of 3‐4 brain sections per animal was calculated.

#### 5.16.11 Co‐Localization Analysis of PSD95 and hNCAM In‐Vitro 3D Constructs

For evaluation of PSD95 expression and co‐localization with hNCAM, immunohistochemistry was completed at 28 days post‐microfluidics fabrication. Constructs were imaged using a Leica TCS SP5 (Leica Microsystems) confocal with a 63x objective and 2.5x zoom using LAS X software. Z‐stacks were acquired with a 1.25 µm step size and a total of 8 slices. A macro was written in Fiji for co‐localization quantification. The background was removed and a gaussian blur (sigma = 1 for PSD95 and sigma = 5 for hNCAM) was applied. Next, the channels were thresholded and the total area was calculated as well as the area overlap between the two channels. The area overlap of PSD95 and hNCAM out of the total hNCAM area was determined.

#### 5.16.12 Co‐Localization of Synaptic proteins‐Axon In Vivo

To assess the presence of synaptic proteins on the implant‐derived axons extending into the mouse cortex, we performed a co‐localization analysis of the pre‐synaptic marker synaptophysin (Syn), and the post‐synaptic marker post synaptic density 95 (PSD95) with the human specific neuronal cell adhesion molecule (NCAM) marker. Images were acquired on a DeltaVision OMX SR microscope system (GE Healthcare) equipped with an Olympus UPlanApo 60x 1.5 NA oil immersion objective, pco.edge sCMOS cameras and 405, 488, 561 and 640 nm lasers. Image stacks were acquired with a z‐distance of 125 nm and with 15 raw images per plane (5 phases, 3 angles). Spherical aberration was corrected using the objective's correction collar. Raw datasets were reconstructed with softWoRx 6.5.2 (GE Healthcare): in particular, a widefield image was obtained, and then deconvolved. Images were auto‐thresholded and converted to 16bit. Color channels were 3D‐registered using Chromagnon 0.85 using a reference image of EdU‐labelled nuclei [78]. To evaluate the colocalization of both pre‐and post‐synaptic proteins onto axons extending from the implant into the adjacent mouse cortex, we utilized the Fiji plugin Synapse Counter [79]. which is a rapid and unbiased analysis pipeline, involving automated identification of particles in separate channels, followed by a comparison of overlapping particles.

### 5.17 Ex vivo Electrophysiology

Five mice were used for these experiments. An average of 3 slices were obtained per animal. Brains were extracted in ice‐cold N‐Methyl‐D‐Glucamine (NMDG)‐based HEPES‐buffered solution, containing in mM: 92 NMDG, 2.5 KCl, 25 glucose, 2 thiourea, 5 sodium ascorbate, 3 sodium pyruvate, 10 MgSO_4_•7H_2_O, 0.5 CaCl_2_•6H_2_O, 1.2 NaH_2_PO_4_•H_2_O, 30 NaHCO_3_, and 20 HEPES (pH 7.4, 300‐310 mOsm/L). Coronal cortical slices (250‐300 µm thick) were cut using a vibratome (Leica VT1200S) and placed in an interface chamber containing artificial cerebrospinal fluid (aCSF) (126 mM NaCl, 3.5 mM KCl, 2 mM MgSO_4_.7H_2_O, 1.25 mM NaH_2_PO_4_, 26 mM NaHCO_3_, 1.2 mM CaCl_2_, and 10 mM glucose; osmolality 300±10 mOsmol/kg) for the duration of the experiment. All solutions were bubbled with carbogen gas (95% O_2_/ 5% CO_2_).

Whole‐cell patch‐clamp recordings were performed using a fixed‐stage upright microscope with oblique illumination (Olympus, BX51WI, 40x/0.8 NA water‐immersion objective). Recordings were obtained with standard borosilicate glass micropipettes (5‐12 MΩ) pulled on a Narishige PC‐10 puller and filled with potassium‐gluconate internal solution (110 mM potassium gluconate; 40 mM HEPES; 4 mM sodium ATP; 2 mM ATP‐Mg, 0.3 mM GTP‐NaCl, 4 mM NaCl, and 4 mg/ml biocytin (Sigma‐Aldrich B4261) with the pH adjusted to 7.2 with KOH and the osmolality 270–290 mOsmol/kg). Epifluorescence imaging using a Cairn OptoLED system was used to identify ChR2‐EYFP+ cells. Electrical signals were amplified with a Multiclamp 700 B amplifier (Molecular Devices, Foster City, CA) and low‐pass filtered and digitized at 10 kHz using an ITC‐18 A/D board (Instrutech) [80]. The recordings were extracted and processed using Igor Pro and later analyzed with custom‐made Python scripts. All statistics were done using GraphPad Prism 10.

### 5.18 Multielectrode Electrophysiology

Brains from the animals were retrieved and the tissue was sliced using the same protocol as the one described for the in vitro whole‐cell patch‐clamp experiments. An average of 3 slices per animal were used for each group. All experiments were performed by experimenters blind to the genotypes of the animals. Recordings were done using a high‐density microelectrode array (MaxOne) from Maxwell Biosystems (Zürich, Switzerland). The MEA provides 26,400 electrodes in a 3.85 × 2.10 mm^2^ large sensing area with an electrode pitch of 17.5 µm [81]. A combination of up to 1,024 electrodes can be recorded from simultaneously. Data is acquired with a sampling rate of 20 kHz [82]. All recordings were 5 min long; each condition was done in triplicate. Baseline recordings were obtained for the first 3 min. Optogenetic stimulation was performed for 1 min. A constant stimulation of the whole of the brain slice was provided for 1 min (Thorlabs, 405 nm, LED, 1000 mA), and then 1 min was left for the slice to recover. Recordings were extracted and pre‐processed using custom‐made Matlab and Python scripts. All statistics were done using GraphPad Prism 10.

### 5.19 Statistical Analysis

Statistical analysis was performed using GraphPad Prism 10. For in vitro 2D culture experiments, 3 biological replicates each one including 6 technical replicates were considered for the 2 week timepoint and 2 biological replicates each one including 4 technical replicates were considered for the 4 week timepoint. The Shapiro‐Wilk normality test was performed for all parameters analyzed. For normally distributed samples, unpaired t‐tests were performed. From all samples analyzed, all but the percentage of SATB2 positive nuclei and the number of degenerating axons were not normally distributed. In this case, a Mann‐Whitney test was performed. For the in vivo cell suspension injection experiments, a Shapiro‐Wilk normality test was performed for all parameters analyzed. Outliers were removed by the Median Absolute Deviation (MAD) method, and unpaired t‐test was performed. Graphs are means and standard error of the mean (SEM): *p<0.05, **p<0.01, ***p <0.001, ****p <0.0001.

## Conflicts of Interest

The authors declare no conflicts of interest.

## Supporting information




**Supporting File 1**: advs73842‐sup‐0001‐SuppMat.docx.

**Supporting File 2**: advs73842‐sup‐0002‐FigureS1.tif.

**Supporting File 3**: advs73842‐sup‐0003‐FigureS2.tif.

**Supporting File 4**: advs73842‐sup‐0004‐FigureS3.tif.

**Supporting File 5**: advs73842‐sup‐0005‐FigureS4.tif.

**Supporting File 6**: advs73842‐sup‐0006‐FigureS5.tif.

**Supporting File 7**: advs73842‐sup‐0007‐FigureS6.tif.

**Supporting File 8**: advs73842‐sup‐0008‐FigureS7.tif.

## Data Availability

The data that support the findings of this study are available from the corresponding author upon reasonable request.
