# Supplementary material for: Astrocyte Enrichment of 3D Cortical Constructs Enhances Brain Repair
Source: Adv Sci (Weinh). 2026 Feb 26;13(20):e07423. doi: 10.1002/advs.202507423 (PMC13067773; doi:10.1002/advs.202507423)
Supplement: Supplementary file 1 — Supporting File 1: advs73842‐sup‐0001‐SuppMat.docx. [file ADVS-13-e07423-s005.docx]

**Supplemental Figures**

**Figure S1. Single cell cultures characterization**

1. Immunofluorescence of primary murine cortical astrocytes at passage 2 cultured in regular astrocyte (mAst) medium and neural maintenance medium NMM, in 2D dimensions (2D). Scale bar=100µm.
2. Conditioned medium experiment. Two weeks in vitro monolayer of cells. NPC only cells were compared with NPCs cultured with conditioned media. Cell culture medium was collected from co-cultured cells (3:1, NPC:murine astrocytes:). The graph shows the mean from n = 3 biological replicates, with statistical analysis done using an unpaired t-test (ns = non-significant).
3. We measured the percentage of area covered by marker immunofluorescence. Neither Nestin nor MAP2 expression was altered by the presence of cultured media. The graph presents the mean from n = 4 biological replicates, analyzed using an unpaired t-test (p < 0.05).
4. Immunofluorescence of mature neurons (MAP2+) in both groups at 4 weeks. Magenta arrowheads point to degenerating axons detected with MAP2. Scale bar=100 μm.
5. The number of degenerating axons decreased in the co-culture group at both 2 and 4 weeks. The graph presents the mean from 4 biological replicates, analyzed using an unpaired t-test (p < 0.05).

**Figure S2. Single-cell suspension implants.**

1. A schematic of the single-cell suspension injections into the motor/somatosensory cortex of postnatal days 7-9 (P7-9) NSG mice.
2. Representative immunofluorescence of brain slices 2 weeks after single cell suspension injection. Low magnification images show 10x tilescan of entire brain section. HuNu immunofluorescence shows location of the cell injection in the somatosensory cortex. Higher magnification images indicate the details and show the high concentration of MAP2+ cells and projections at the injection site. NeuN immunofluorescence was obvious in the mouse cortex, but only a few were detected in the implant. Scale bar=1mm in the lower magnification images. Scale bar=100µm in higher magnification images.
3. Immunofluorescence of cells implanted in the mouse cortex 2 weeks post-injection. Human neural progenitor cells (NPCs) were identified with HuNu antibodies, while NeuN marked mature neurons, Ki67 indicated proliferation, and activated caspase-3 (aCasp3) signified cell death. Scale bar=50 μm.
4. Quantification of NeuN+ as a proportion of all HuNu+ cells. The graph displays the mean across (n = 5 mice) biological replicates for the NPC group and (n = 6 mice) for the co-culture group, with statistical significance assessed using an unpaired t-test (p < 0.001).
5. Quantification of double positive nuclei for Ki67 and HuNu. The graph shows the mean across (*n* = 5 mice) biological replicates for the NPC group and (*n* = 6 mice) for the co-culture group, with statistical significance assessed using an unpaired t-test (p < 0.0001)
6. Graph representing quantification of aCas3+ as a proportion of all HuNu+ cells. The graph displays the mean across (*n* = 5 mice) biological replicates for the NPC group and (*n* = 6 mice) for the co-culture group, with statistical significance assessed using an unpaired t-test (p = 0.0673).

**Figure S3. Murine astrocytes survive in neural progenitor media in 2D and 3D.**

1. Representative images of microfluidics-generated constructs containing only primary murine cortical astrocytes, at 2 and 4 weeks post-assembly, immunostained for GFAP and S100b. Scale bar=100µm.
2. There was no significant difference between the NPC and co-culture groups in the area occupied by Nestin expression. Data represent the mean of three biological replicates (n=3). Statistical significance was tested using an unpaired t-test (ns = non-significant).
3. There was no significant difference between the NPC and co-culture groups in the area occupied by Nestin or the percent of cells that expressed CTIP2. Data represent the mean of three biological replicates (n=3). Statistical significance was tested using an unpaired t-test (ns = non-significant).

**Figure S4. Co-culture 3D constructs are smaller.**

(A) Representative images of NPC only and co-culture constructs at 3 DPA in vitro showing size difference between the groups. Scale bar=500µm.

(B) The relative area and the total volume of the NPC only group was greater in comparison with co-culture constructs in vitro at 3 DPA. Graphs represent means across 7 constructs for each group for relative area and 3 constructs for each group for volume, with statistical significance assessed using an unpaired t-test (p<0.01; p<0.0001).

(C) Representative Neurolucida 3D images of NPC only and co-culture microfluidics-generated constructs at 3, 14 and 28 DPA in vitro. Traces represent slices measurement used for volume calculation. Scale bar=500µm.

(D) Comparison of the volume of the NPC only and co-culture groups showed no statistical difference at 14 and 28 DPA. Graphs represent means across 5 constructs for NPC group and 4 constructs for co-culture group at 14 DPA and 3 constructs at 28 DPA. Significance was assessed using an unpaired t-test. (ns=non-significant).

(E) Analysis of the relative growth of the constructs in both groups showed that in the co-culture group, constructs had an increased growth rate at 14 DPA, which was not seen at 28 DPA. Graphs represent means across 5 constructs for NPC only group and 4 constructs for the co-culture group at 14 DPA and 3 constructs at 28 DPA. Statistical significance was assessed using an unpaired t-test (p<0.05).

(F) Representative immunofluorescence images of NPC and co-culture constructs in vitro at 14 DPA. Scale bar=50µm.

(G) Graph representing the percent of NeuN+ nuclei in the constructs showed no statistical difference in the NPC only and co-culture group. Graphs represent means across 3 constructs for the NPC only group and 2 constructs for the co-culture group. This was assessed using an unpaired t-test.

(H) Graph representing percentage of Ki67+ nuclei in the NPC and co-culture groups at 14 DPA. Graphs represent means across 3 constructs for NPC only group and 2 constructs for co-culture group. Statistical assessment was done using an unpaired t-test.

**Figure S5. GFAP+ cells complexity between groups.**

(A) Morphological profiling of astrocytes by Scholl analysis indicated no statistical difference between the two groups for the area and total intersection number parameters. Astrocytes in the co-culture group present increased Scholl decay when compared to NPC only group. Graphs represent means across 27 cells in 5 animals per group. Statistical assessment was done using an unpaired t-test (p<0.05).

(B) Comparison of the morphology of astrocytes in the contralateral cortex of NPC only and co-culture implanted animals. Statistical analysis showed decreased area and increased Sholl decay for the co-culture group. Graphs represent means for 21 and 22 cells across 3 animals for the NPC only and co-culture groups, respectively. Statistical assessment was done using an unpaired t-test (p<0.01).

(C) Representative figure showing the Manders' Colocalization Coefficient calculation using the Biop JacoP plugin on Fiji ImageJ. The fraction of GFAP over S100β (M1) and S100β over GFAP (M2) was calculated in both groups. Graphs show means with SEM. ns: non-significant. Scale bar=100µm.

**Figure S6. Neuronal markers, proliferation and GFAP lesion border are not different between groups.**

(A) Representative immunofluorescence for GFAP of brain section 56 DP TBI, with image of the whole brain surface, showing the TBI lesion. Scale bar=500µm.

(B) Representative immunofluorescence for GFAP in the lesion border of TBI (no implant), NPC only and co-culture groups at 56 DP TBI. The average fluorescence intensity profile across 3 lines per image, represented by dotted lines, was measured for each group. Scale bar=500µm.

(C) Shows the Normalized Grey Value of GFAP for TBI only, NPC and co-culture. The number of GFAP peaks and the area under each curve was measured and is shown.

**Figure S7. AAV Plasmid map and labeled cell.**

1. Sequence of AAV obtained from Add Gene used in electrophysiology studies.
2. YFP fluorescence in neuron found in NPC construct.
