# Supplementary figures and images for: Astrocyte Enrichment of 3D Cortical Constructs Enhances Brain Repair
Source: Adv Sci (Weinh). 2026 Feb 26;13(20):e07423. doi: 10.1002/advs.202507423 (PMC13067773; doi:10.1002/advs.202507423)

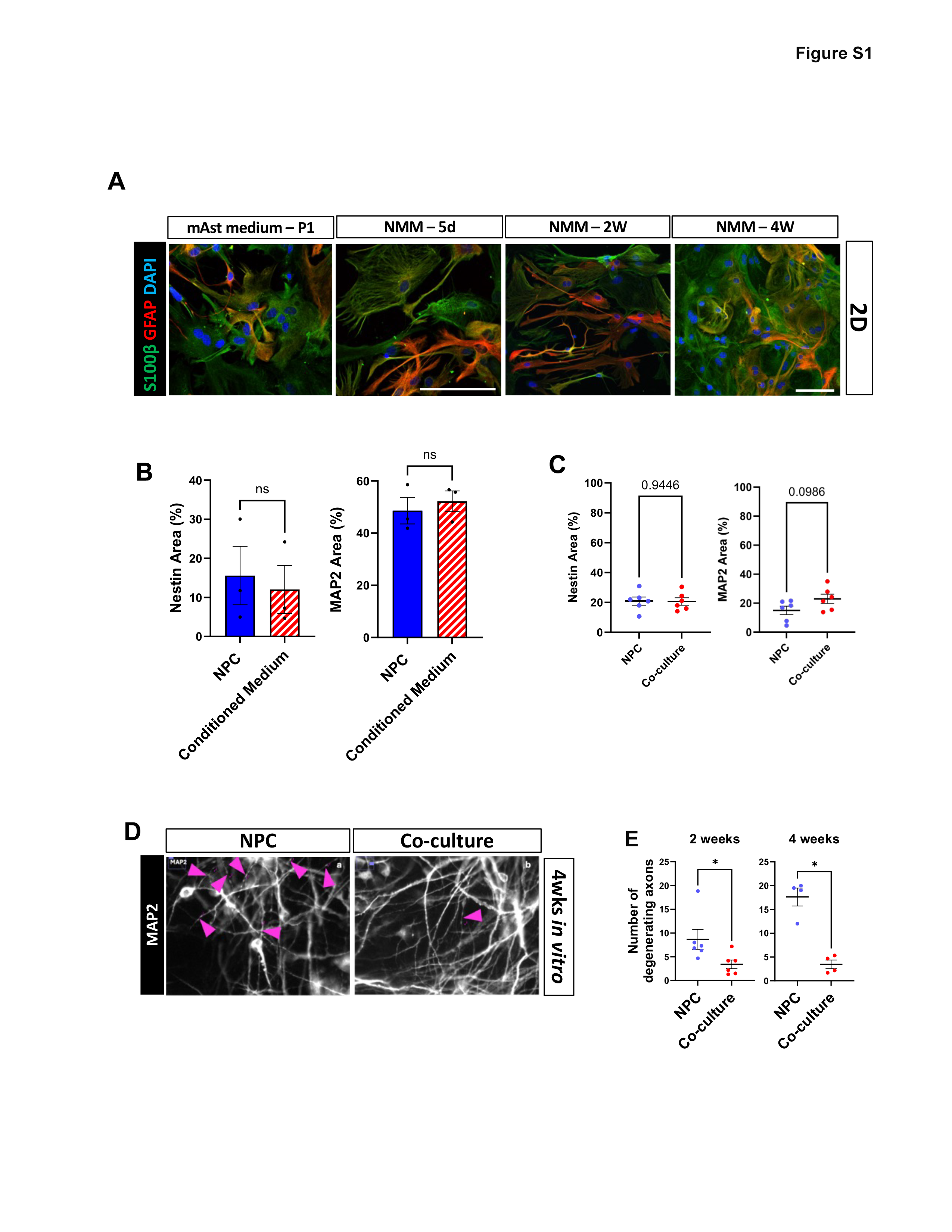

Supplement: Supplementary file 2 — Supporting File 2: advs73842‐sup‐0002‐FigureS1.tif. [file ADVS-13-e07423-s004.tif]

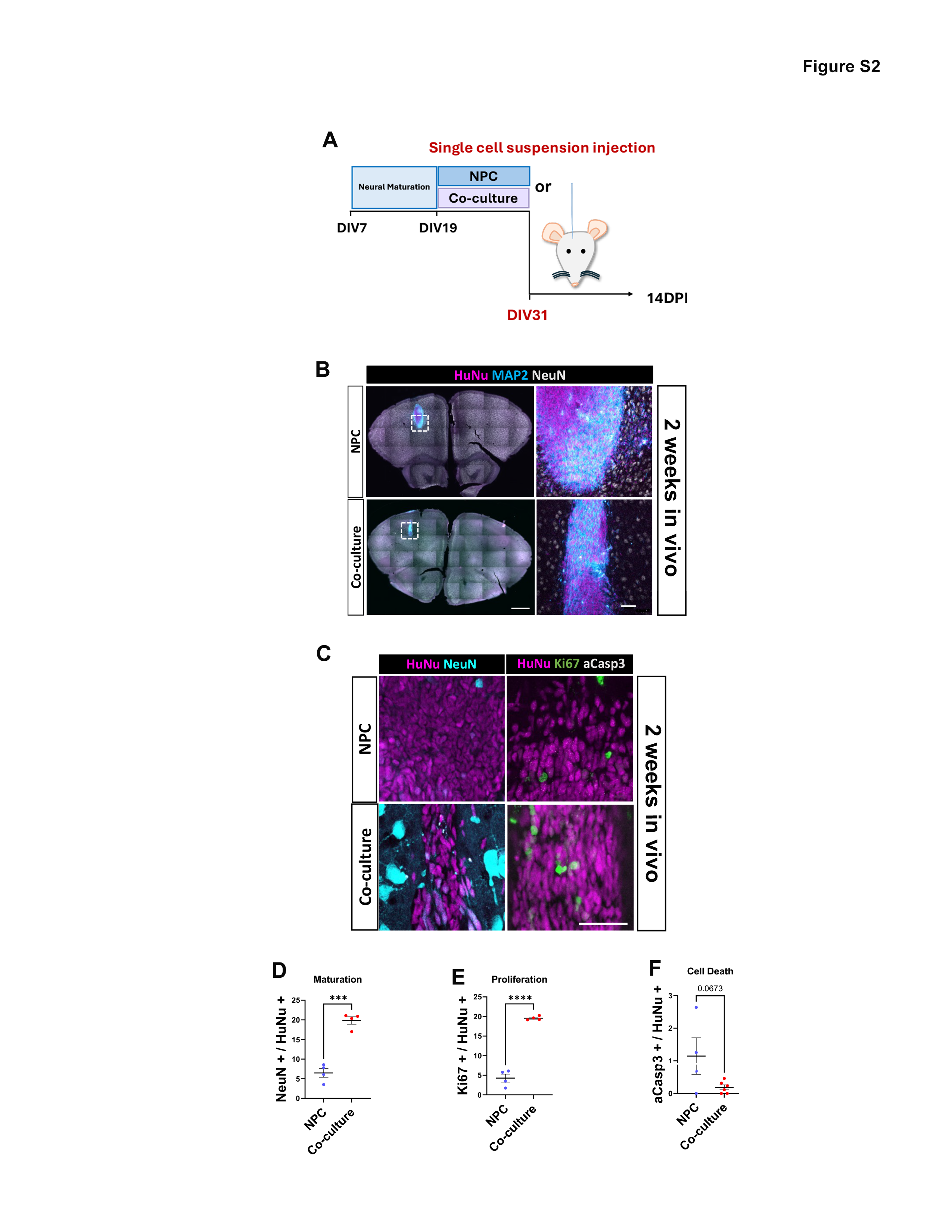

Supplement: Supplementary file 3 — Supporting File 3: advs73842‐sup‐0003‐FigureS2.tif. [file ADVS-13-e07423-s003.tif]

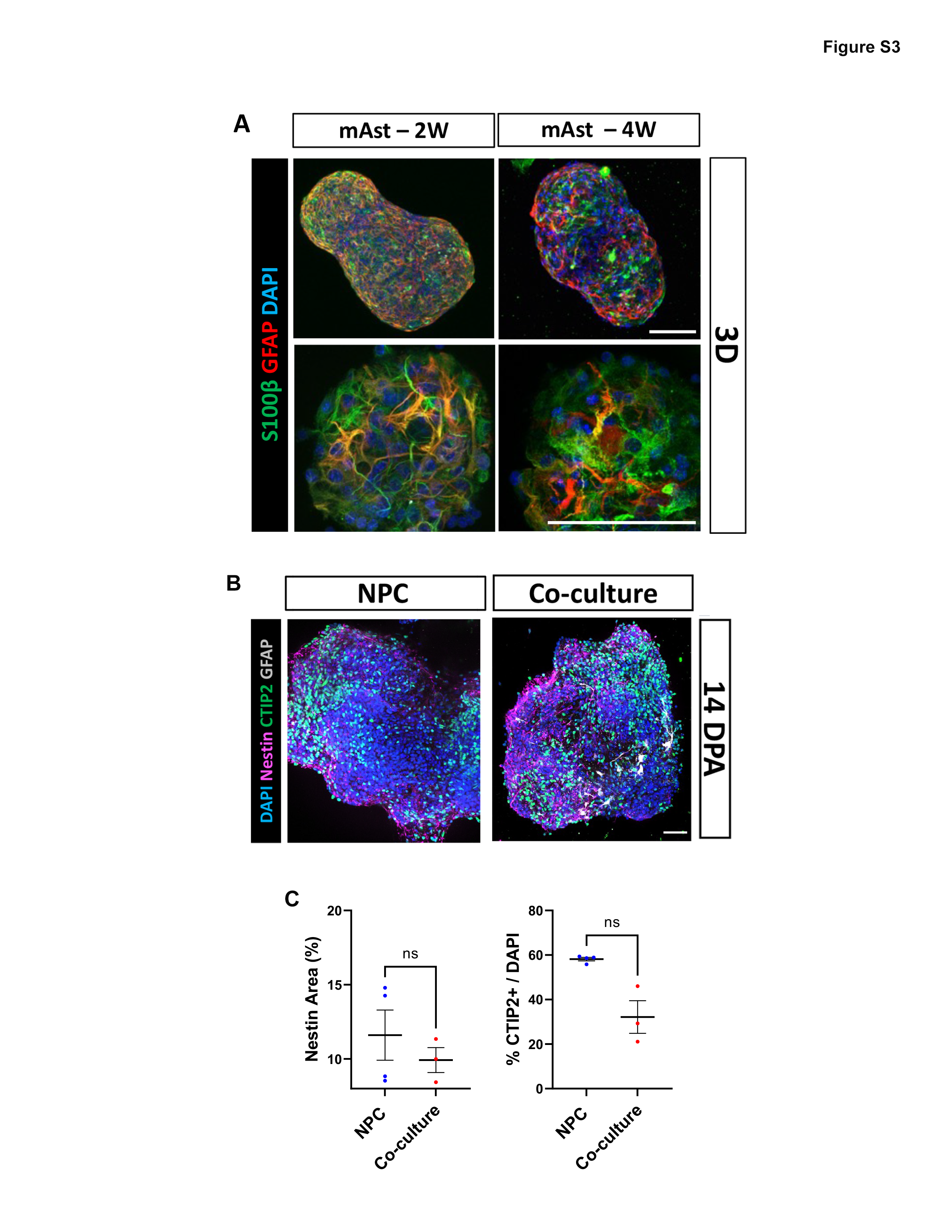

Supplement: Supplementary file 4 — Supporting File 4: advs73842‐sup‐0004‐FigureS3.tif. [file ADVS-13-e07423-s001.tif]

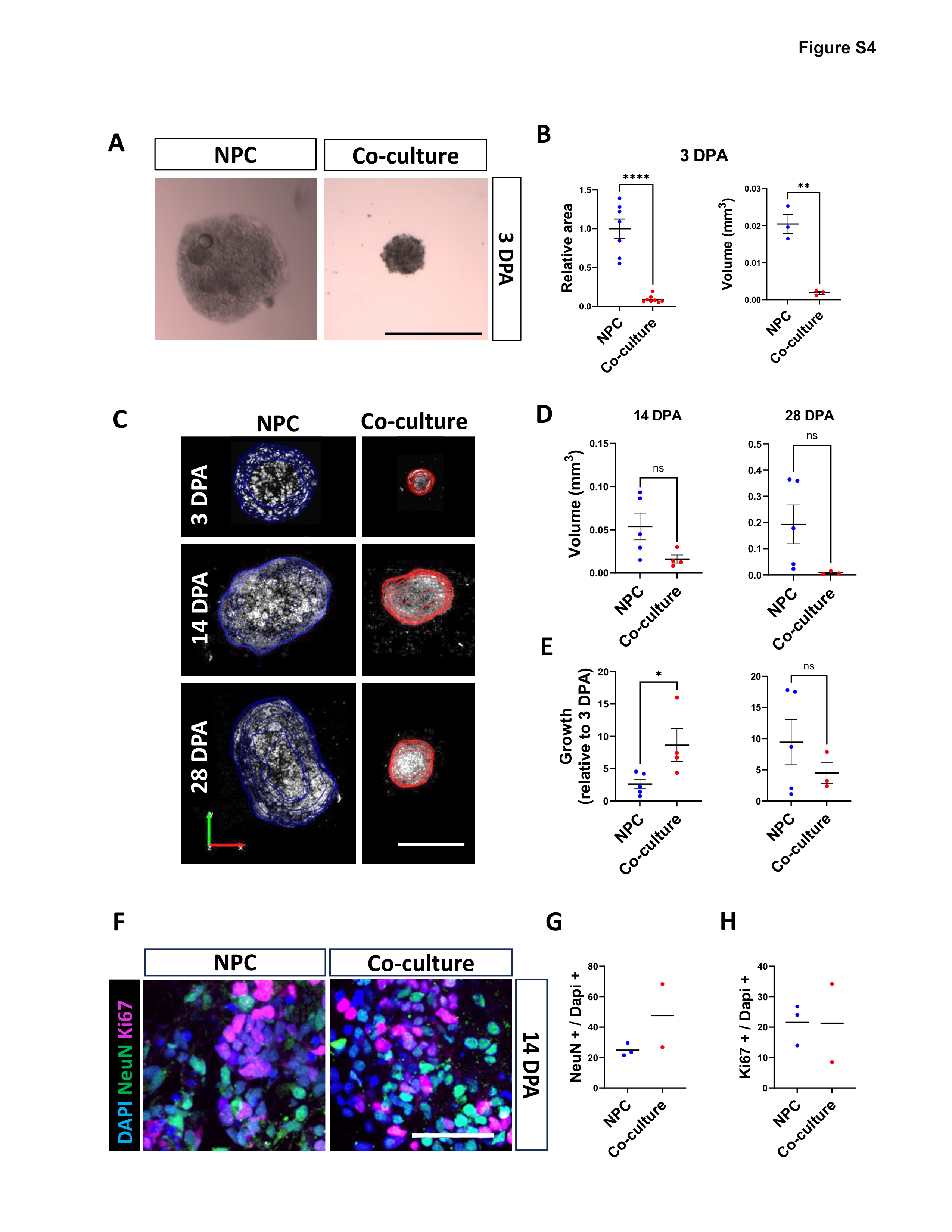

Supplement: Supplementary file 5 — Supporting File 5: advs73842‐sup‐0005‐FigureS4.tif. [file ADVS-13-e07423-s007.tif]

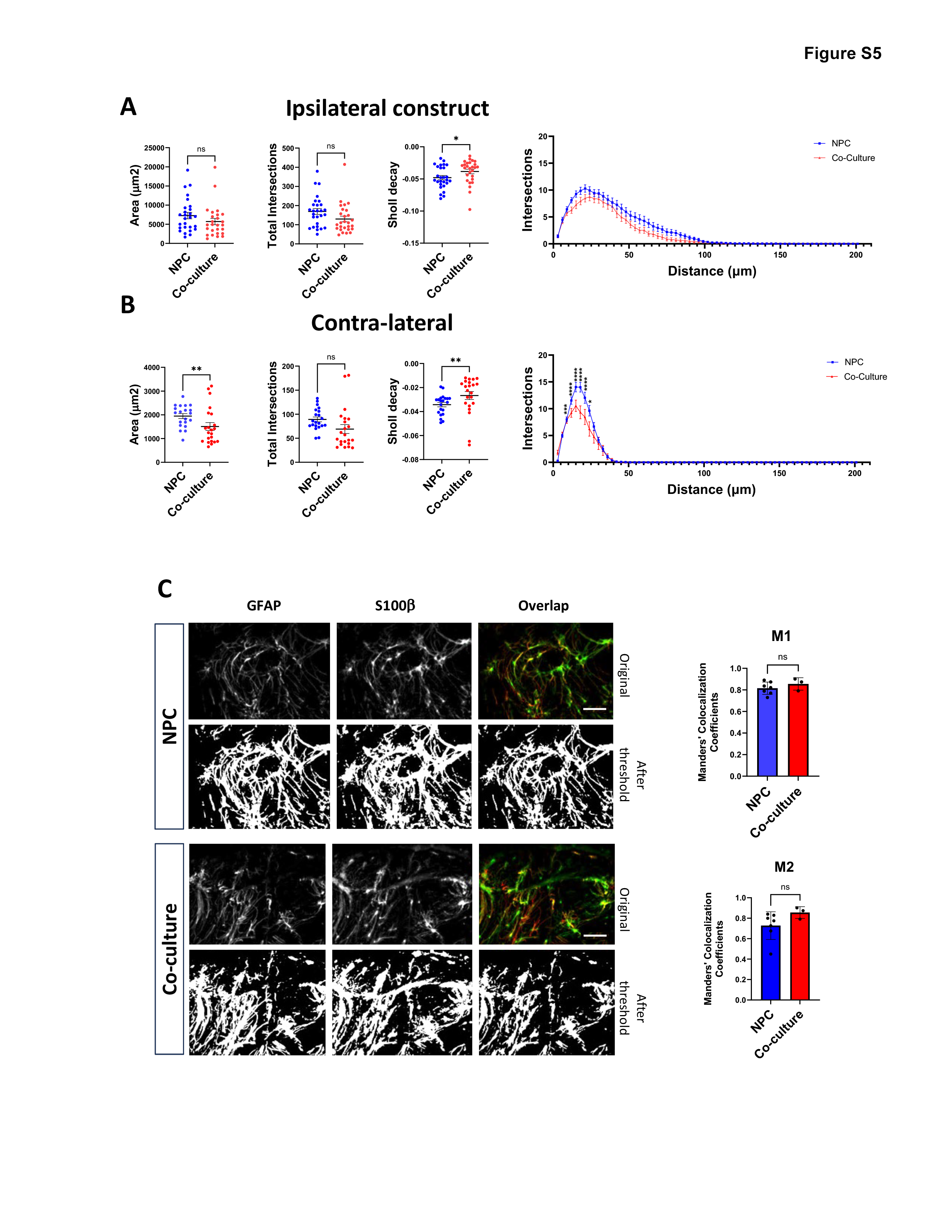

Supplement: Supplementary file 6 — Supporting File 6: advs73842‐sup‐0006‐FigureS5.tif. [file ADVS-13-e07423-s002.tif]

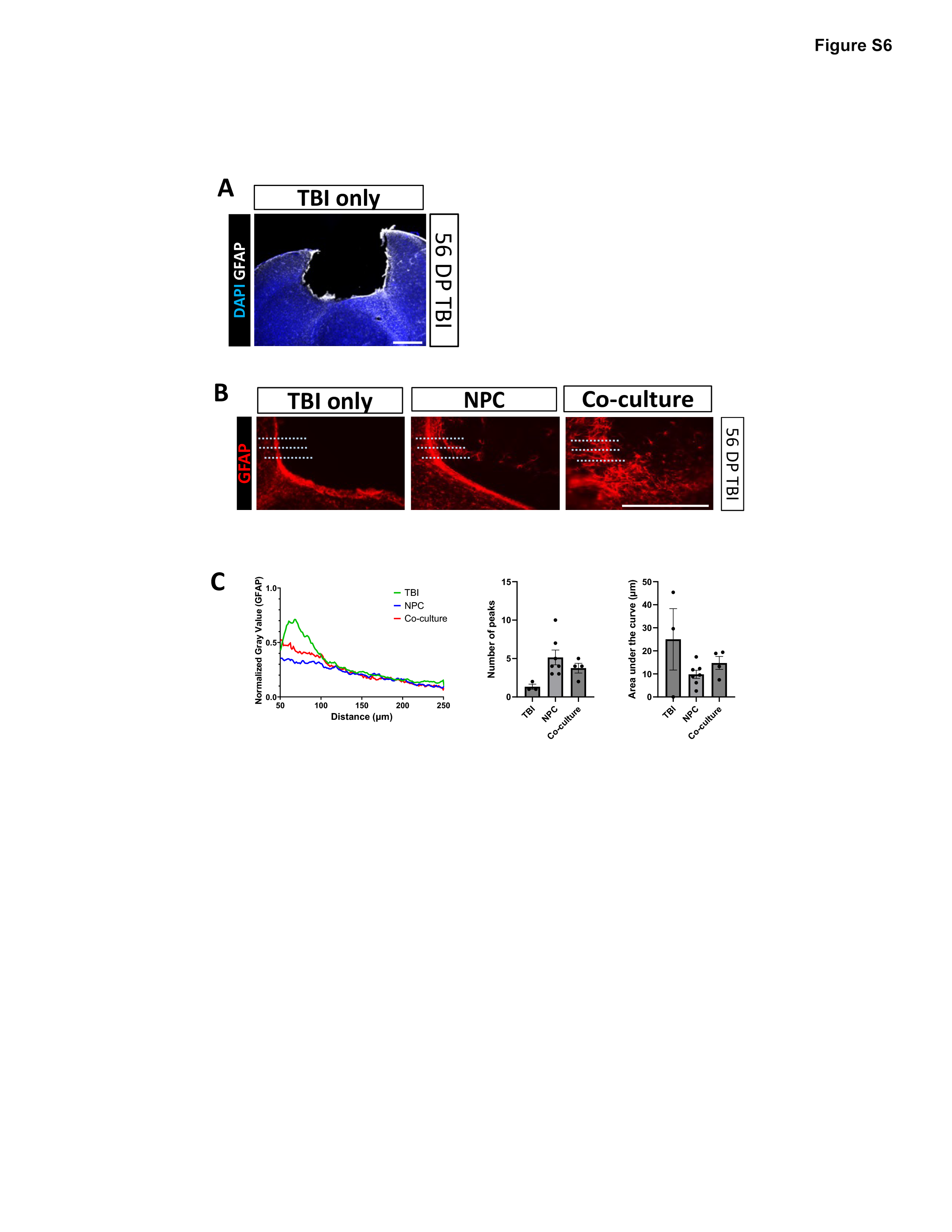

Supplement: Supplementary file 7 — Supporting File 7: advs73842‐sup‐0007‐FigureS6.tif. [file ADVS-13-e07423-s008.tif]

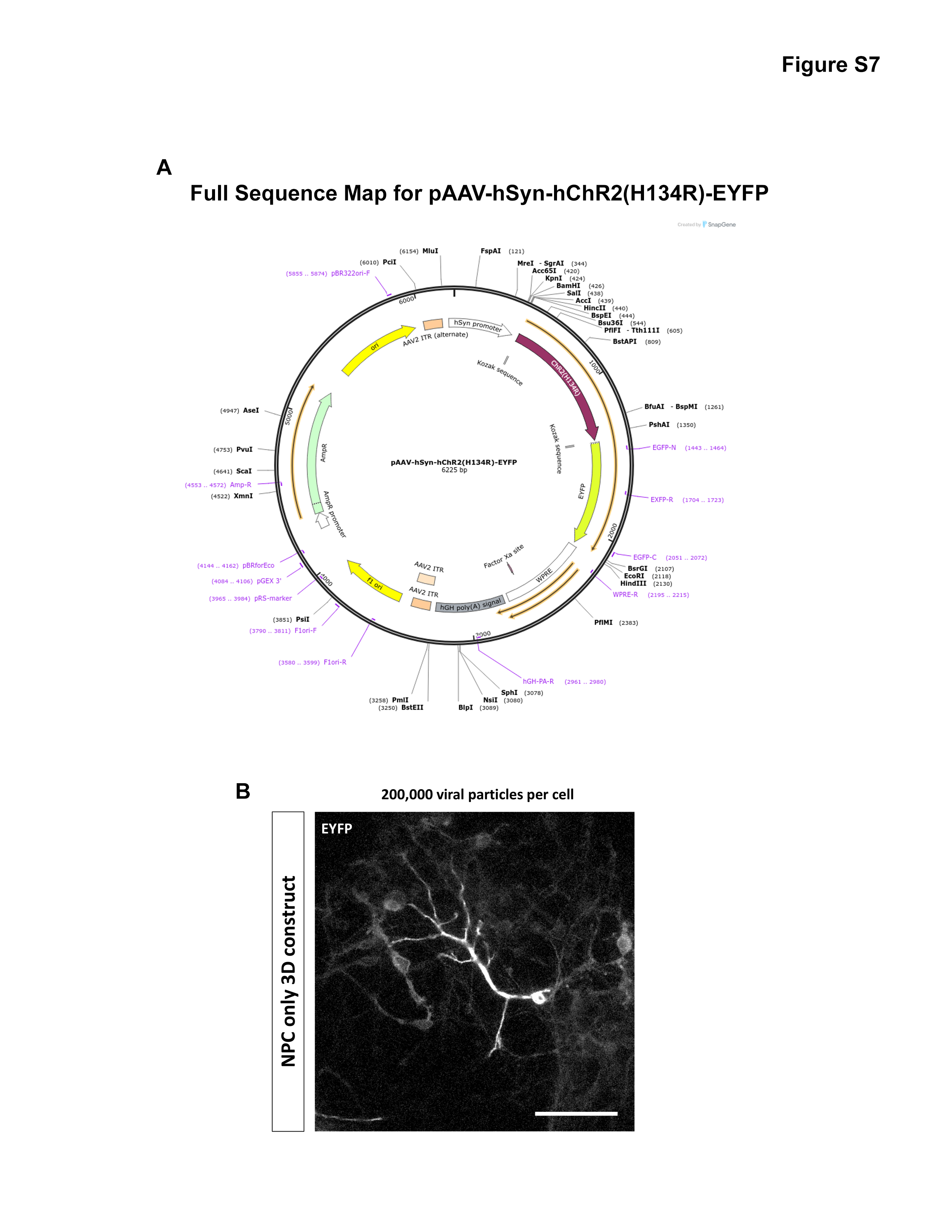

Supplement: Supplementary file 8 — Supporting File 8: advs73842‐sup‐0008‐FigureS7.tif. [file ADVS-13-e07423-s006.tif]
